# Energy landscape decomposition for cell differentiation with proliferation effect

**DOI:** 10.1093/nsr/nwac116

**Published:** 2022-06-17

**Authors:** Jifan Shi, Kazuyuki Aihara, Tiejun Li, Luonan Chen

**Affiliations:** Research Institute of Intelligent Complex Systems, Fudan University, Shanghai 200433, China; International Research Center for Neurointelligence, The University of Tokyo Institutes for Advanced Study, The University of Tokyo, Tokyo 113-0033, Japan; International Research Center for Neurointelligence, The University of Tokyo Institutes for Advanced Study, The University of Tokyo, Tokyo 113-0033, Japan; Key Laboratory of Mathematics and Its Applications (LMAM) and School of Mathematical Sciences, Peking University, Beijing 100871, China; Key Laboratory of Systems Biology, Shanghai Institute of Biochemistry and Cell Biology, Center for Excellence in Molecular Cell Science, Chinese Academy of Sciences, Shanghai 200031, China; Key Laboratory of Systems Health Science of Zhejiang Province, Hangzhou Institute for Advanced Study, University of Chinese Academy of Sciences, Chinese Academy of Sciences, Hangzhou 310024, China; School of Life Science and Technology, ShanghaiTech University, Shanghai 201210, China; Guangdong Institute of Intelligence Science and Technology, Zhuhai 519031, China

**Keywords:** energy landscape, cell differentiation, stochastic systems

## Abstract

Complex interactions between genes determine the development and differentiation of cells. We establish a landscape theory for cell differentiation with proliferation effect, in which the developmental process is modeled as a stochastic dynamical system with a birth-death term. We find that two different energy landscapes, denoted *U* and *V*, collectively contribute to the establishment of non-equilibrium steady differentiation. The potential *U* is known as the energy landscape leading to the steady distribution, whose metastable states stand for cell types, while *V* indicates the differentiation direction from pluripotent to differentiated cells. This interpretation of cell differentiation is different from the previous landscape theory without the proliferation effect. We propose feasible numerical methods and a mean-field approximation for constructing landscapes *U* and *V*. Successful applications to typical biological models demonstrate the energy landscape decomposition's validity and reveal biological insights into the considered processes.

## Teaser

Energy landscape decomposition (ELD) theory provides a computational tool to construct epigenetic and pluripotency landscapes for cell differentiation process with proliferation effect.

## INTRODUCTION

Cell differentiation makes the biological world rich and colorful. Modeling and understanding the dynamics of gene regulatory networks (GRNs) are essential to explore the underlying mechanism of cell differentiation [1]. Since Waddington proposed his seminal metaphor of the epigenetic landscape [2,3], differentiation has been intuitively described as a ball rolling down a surface, i.e. the energy landscape.

There are mainly two approaches to construct the energy landscape for biosystems. One is the data-based approach, which tries to identify cluster/cell types, differentiation trajectories, pseudotime and cell pluripotency from the experimental data, especially single-cell RNA sequencing (scRNA-seq) data [4–8]. Various algorithms have been proposed from the theory of graph [9,10], entropy [6,11,12] and dynamical systems [13–16]. Among these data-based methods, the population balance analysis (PBA) [13–15] and landscape of differentiation dynamics (LDD) [16] especially involve cell proliferation and death rates, which generalize the hypothesis of cell differentiation from equilibrium to non-equilibrium steady processes. The other approach is the model-based approach, which tries to build dynamic equations from the GRN, analyze the dynamical behavior of the system and then construct energy landscapes using numerical simulations. Wang *et al.* proposed a practical framework for constructing energy landscapes for biosystems without cell birth and death effects [17–19]. They defined the landscape as $U(\boldsymbol{x}) = -\epsilon \log P_{ss}(\boldsymbol{x})$, where $\boldsymbol{x}$ is the gene expression vector, $P_{ss}(\boldsymbol{x})$ is the steady probability density function (PDF) of the system and ε is related to the amplitude of small intrinsic noise. By changing parameter values to control the biological process, the landscape pattern varies, which gives an intuitive description of cell-type locations and transition probability between clusters. This framework has been widely used in modeling the budding yeast cell cycle [17], human stem cell fate [19] and *Caenorhabditis elegans* ageing [20]. Without the cell proliferation effect, however, the direction of differentiation is usually not intrinsic in Wang’s landscape but controlled by manually setting parameters. Beyond the topics above, there are also many other interesting works related to the energy landscape theory [21–27].

In this study, we follow the model-based approach but consider the birth and death rates (BDRs) of cells in differentiation, which is inspired by the PBA and LDD. We show that there are two important energy landscapes to describe differentiation dynamics, which are denoted $U(\boldsymbol{x})$ and $V(\boldsymbol{x})$ and are also computable using the model. The metastable states in $U(\boldsymbol{x})$ represent cell types, and the value of $V(\boldsymbol{x})$ implicates pluripotency. The negative gradient of $V(\boldsymbol{x})$ shows the direction of differentiation. Taking BDRs into account is essential for constructing the pluripotency landscape $V(\boldsymbol{x})$. Here, we explain our theory of the energy landscape decomposition (ELD) in cell differentiation with proliferation effect. Numerical algorithms for constructing energy landscapes and the mean-field approximation (MFA) in high-dimensional cases are also proposed. We use three examples to show the application of the ELD, where $U(\boldsymbol{x})$ and $V(\boldsymbol{x})$ intuitively explain the processes of cell differentiation.

## RESULTS

### Modeling cell differentiation at the population level

Motivated by Weinreb *et al.* [13] and Briggs *et al.* [15], we modeled cell development through a stochastic dynamical system with the birth-death term. We denote gene expression levels using $\boldsymbol{x}\in \mathbb {R}^n$. During cell differentiation, the cell population is quantified using a PDF $p(\boldsymbol{x}, t)$, whose evolution follows a generalized form of the well-known Fokker-Planck equation (FPE), i.e.

(1) \begin{eqnarray*} \frac{\partial p(\boldsymbol{x}, t)}{\partial t} &=& -\nabla \cdot (\boldsymbol{b}(\boldsymbol{x})p(\boldsymbol{x}, t)) + \epsilon \Delta p(\boldsymbol{x}, t)\nonumber\\ && +\, R(\boldsymbol{x})p(\boldsymbol{x}, t), \end{eqnarray*}

with an initial PDF $p(\boldsymbol{x}, t=0)$ at *t* = 0, where $\boldsymbol{b}(\boldsymbol{x})$ represents the biological interactions between genes, ε is a parameter standing for the noise amplitude and $R(\boldsymbol{x})$ stands for the net BDR of cells at $\boldsymbol{x}$. In a practical application, $\boldsymbol{b}(\boldsymbol{x})$ is usually modeled using either activated or inhibited Hill functions, according to the considered GRN. Here $R(\boldsymbol{x})>0$ indicates the proliferation of cells, while $R(\boldsymbol{x}) <0$ indicates the death of cells. When $R(\boldsymbol{x}) \equiv 0$, (1) reduces to the standard FPE without any cell proliferation effect. To ensure a non-explosive and non-degenerative steady PDF $p_{ss}(\boldsymbol{x})$ for (1), we require the following condition as a constraint for $R(\boldsymbol{x})$:

(2) \begin{equation*} \int R(\boldsymbol{x})p_{ss}(\boldsymbol{x})\mathop {}\!\mathrm{d}\boldsymbol{x} = 0. \end{equation*}

It is necessary to have a non-trivial $R(\boldsymbol{x})$ to characterize the population balance of the cell birth and death in the steady state.

### Modeling cell differentiation at the single-cell level

The population-level dynamics in (1) can also be viewed as the probabilistic interpretation at the single-cell level. We consider a cell ω with gene expression $\boldsymbol{X}_t(\omega )$ starting from $\boldsymbol{Y}_0$ at *t* = 0 and propose a weighted stochastic dynamics in the Itô sense as

(3a) \begin{equation*} \mathop {}\!\mathrm{d}\boldsymbol{X}_t(\omega ) = \boldsymbol{b}(\boldsymbol{X}_t(\omega ))\mathop {}\!\mathrm{d}t + \sqrt{2\epsilon } \mathop {}\!\mathrm{d}\boldsymbol{W}_t(\omega ), \end{equation*}

(3b) \begin{equation*} \boldsymbol{X}_t|_{t=0} = \boldsymbol{Y}_0(\omega ), \end{equation*}

(3c) \begin{equation*} \mathop {}\!\mathrm{d}\rho _t(\omega ) = R(\boldsymbol{X}_t(\omega ))\rho _t(\omega )\mathop {}\!\mathrm{d}t, \end{equation*}

(3d) \begin{equation*} \rho _t|_{t=0} = 1, \end{equation*}

where $\boldsymbol{Y}_0$ is distributed according to $p_0(\boldsymbol{x})$, $\boldsymbol{W}_t$ is a standard Brownian motion with independent components and ρ_*t*_(ω) stands for a time-varying weight for cell ω. The connection between (1) and (3) is represented as

(4) \begin{equation*} p(\boldsymbol{x}, t) = \mathbb {E}\lbrace \rho _t(\omega )\delta (\boldsymbol{x}-\boldsymbol{X}_t(\omega ))\rbrace , \end{equation*}

where δ is the Dirac delta function and the expectation is taken over all the possible trajectories ω (see the online supplementary material for details). The weight ρ_*t*_(ω) increases at *t* when $R(\boldsymbol{X}_t(\omega ))>0$, which corresponds to cell proliferation, while it decreases at *t* when $R(\boldsymbol{X}_t(\omega ))<0$, which corresponds to cell death. If $R(\boldsymbol{x})\equiv 0$, the weight ρ_*t*_(ω) is a constant for every cell and the system reduces to the case without any cell proliferation, which is discussed by Wang *et al.* [17,18].

### Energy landscape decomposition

Based on the above dynamical modeling of cell development, we focus on the construction of the landscapes using the model-based approach, i.e. we assume that $\boldsymbol{b}(\boldsymbol{x}), \epsilon$, and $R(\boldsymbol{x})$ are known a priori.

We denote by $P_U(\boldsymbol{x})$ the steady PDF with the known BDR $R(\boldsymbol{x})$ and by $P_0(\boldsymbol{x})$ the steady PDF with $R(\boldsymbol{x})\equiv 0$, for (1). Note that the notation $P_0(\boldsymbol{x})$ is different from the initial distribution $p(\boldsymbol{x}, t=0)$. Then, two energy landscapes are constructed as

(5) \begin{equation*} U(\boldsymbol{x}) = -\epsilon \log P_U(\boldsymbol{x}), \end{equation*}

which drives the system or cells to the steady distribution, and

(6) \begin{eqnarray*} V(\boldsymbol{x}) &=& -\epsilon \log P_0(\boldsymbol{x}) - (-\epsilon \log P_U(\boldsymbol{x}))\nonumber \\ &=& -\epsilon \log (P_0(\boldsymbol{x})/P_U(\boldsymbol{x})), \end{eqnarray*}

which quantifies the change of the potential caused by $R(\boldsymbol{x})$, i.e. the influence induced by cell proliferation and death. The metastable basins in landscape $U(\boldsymbol{x})$ indicate cell types, and the depth of each basin characterizes its stability. The values of *V* depict the pluripotency and its negative gradient field describes the differentiation direction. From a stem cell state to a differentiated cell state, *V* decreases gradually. The potential *U* is the cell-type landscape and *V* is the pluripotency landscape. Besides the two potential functions, the remaining term $\boldsymbol{f}(\boldsymbol{x})$is defined as

(7) \begin{equation*} \boldsymbol{f}(\boldsymbol{x}) = \boldsymbol{b}(\boldsymbol{x}) + \nabla U(\boldsymbol{x}) + \nabla V(\boldsymbol{x}), \end{equation*}

which is the curl part that describes the non-gradientness of the considered dynamics. The $\boldsymbol{f}(\boldsymbol{x})$ term satisfies

(8) \begin{equation*} \nabla \cdot (\boldsymbol{f}(\boldsymbol{x})P_0(\boldsymbol{x})) = 0, \end{equation*}

which corresponds to the divergence-free condition of the curl flux $\boldsymbol{J}\!\!(\boldsymbol{x})=\boldsymbol{f}(\boldsymbol{x})P_0(\boldsymbol{x})$ defined in [17,18].

In summary, we propose an energy decomposition for the differentiation dynamics characterized by the pair $(\boldsymbol{b}(\boldsymbol{x}), R(\boldsymbol{x}))$ in our ELD framework as

(9) \begin{equation*} \boldsymbol{b}(\boldsymbol{x}) = -\nabla U(\boldsymbol{x}) - \nabla V(\boldsymbol{x}) + \boldsymbol{f}(\boldsymbol{x}). \end{equation*}

**Figure 1.**
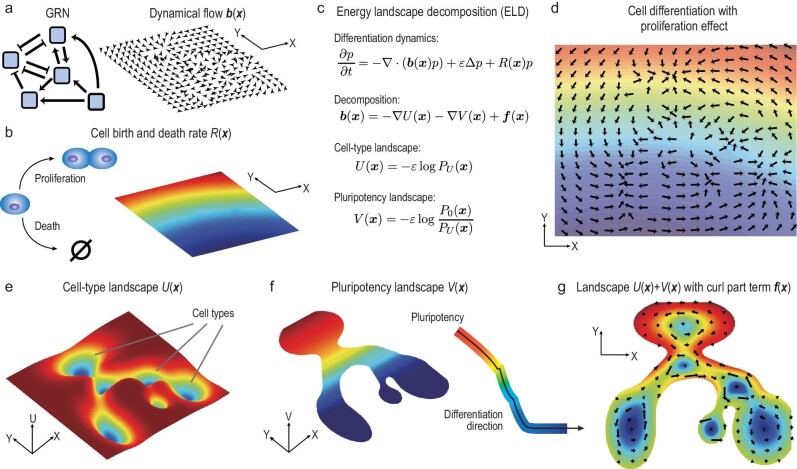
An illustration of the ELD framework. (a) The GRN, which reins cell differentiation dynamics. Dynamical flow $\boldsymbol{b}(\boldsymbol{x})$ can be modeled using the GRN, and its vector field is drawn in an illustrative space (*X*, *Y*). (b) Cells can proliferate or die at a natural birth and death rate $R(\boldsymbol{x})$. Proliferation with $R(\boldsymbol{x})>0$ is shown in red, while cell death with $R(\boldsymbol{x})<0$ is in blue (jet colormap). (c) The ELD theory in this study. The dynamics $(\boldsymbol{b}(\boldsymbol{x}), R(\boldsymbol{x}))$ can be characterized using two potential terms (energy landscapes) $U(\boldsymbol{x})$ and $V(\boldsymbol{x})$, and a curl part term $\boldsymbol{f}(\boldsymbol{x})$. We define $U(\boldsymbol{x})$ and $V(\boldsymbol{x})$ using two steady PDFs, denoted $P_U(\boldsymbol{x})$ and $P_0(\boldsymbol{x})$. (d) The combination of dynamical flow $\boldsymbol{b}(\boldsymbol{x})$ (arrows) and birth and death rate $R(\boldsymbol{x})$ (jet colored background). (e–g) Illustrations for the cell-type landscape $U(\boldsymbol{x})$, the pluripotency landscape $V(\boldsymbol{x})$ and the curl part term $\boldsymbol{f}(\boldsymbol{x})$, respectively. Metastable states of $U(\boldsymbol{x})$ shown in (e) stand for different cell types. The values of $V(\boldsymbol{x})$ shown in (f) indicate the pluripotency, with its negative gradient depicting the differentiation direction. The color bar in (f) is calculated using the mean value of *V* along the *X* axis, which displays an intuitive global differentiation direction. In (g), landscape $U(\boldsymbol{x})+V(\boldsymbol{x})$ is plotted as the background, while the curl part term $\boldsymbol{f}(\boldsymbol{x})$ is indicated by the vectors.

The potentials *U* and *V*, together with the curl part $\boldsymbol{f}$, help us have a deep understanding of cell differentiation and cell subtypes along the differentiation pathway. Figure 1 presents an illustration of the whole ELD procedure.

The connection between the ELD framework and the existing landscape theory is as follows. In the case of no birth and death of cells, i.e. $R(\boldsymbol{x})\equiv 0$, (9) reduces to Wang’s landscape decomposition [17–19] with $P_U(\boldsymbol{x})\equiv P_0(\boldsymbol{x})$ and $V(\boldsymbol{x})\equiv 0$; if the system is of gradient type with $\boldsymbol{b}(\boldsymbol{x}) = -\nabla F(\boldsymbol{x})$, (9) reduces to the landscape decomposition discussed in PBA and LDD [13,16] with $\boldsymbol{f}(\boldsymbol{x})\equiv 0$.

In a case that only $p_{ss}(\boldsymbol{x})$ is known, PBA and LDD define the energy landscape *V* for gradient systems by solving the equation

(10) \begin{equation*} \nabla \log p_{ss}(\boldsymbol{x})\cdot \nabla V(\boldsymbol{x}) + \Delta V(\boldsymbol{x}) = -R(\boldsymbol{x}). \end{equation*}

with proper boundary conditions. However, for a non-gradient system, we show that (10) is not always valid, unless the curl part $\boldsymbol{f}$ satisfies $\boldsymbol{f}(\boldsymbol{x})\perp \nabla V(\boldsymbol{x})$ almost everywhere (see the online supplementary material for details). In this sense, we provide a more general definition of *V* using (6). More detailed discussion about the ELD can be found in the online supplementary material.

### Numerical construction of energy landscapes

According to the definitions of potentials $U(\boldsymbol{x})$ in (5) and $V(\boldsymbol{x})$ in (6), we can compute the energy landscapes once the steady distributions $P_U(\boldsymbol{x})$ and $P_0(\boldsymbol{x})$ are obtained. Next we discuss the numerical methods for the low-dimensional case and the high-dimensional case separately.

#### Solving FPE for the low-dimensional case

For the low-dimensional case (usually in less than three dimensions), we used the finite difference or finite element method to solve (1) numerically until the steady state. For a small parameter ε, the spatial scale *h* of the grids in traditional methods is needed to be set as *h* ≪ ε to resolve the dynamical behavior of such a convection-dominated equation, which would be impractical. We utilized the streamline diffusion method [28] that can avoid the numerical oscillations even when *h* > ε. The detailed procedure of the streamline diffusion method is described in the online supplementary material in a two-dimensional case. It is also applied in our numerical examples in the Results section.

#### Mean-field approximation in the high-dimensional case

For the high-dimensional case, it is not feasible to directly solve (1). Thus, we start from the single-cell dynamics (3) and propose an MFA approach to reduce the computational complexity from exponential to polynomial time. Overall, the potentials *P*_*U*_ and *P*_0_ are approximated by the Gaussian mixtures in the MFA, i.e.

(11) \begin{equation*} p(\boldsymbol{x}) = \sum _{k=1}^K\rho ^{(k)}p_{(k)}(\boldsymbol{x}; \boldsymbol{\mu }_{(k)}, \boldsymbol{\Sigma }_{(k)}), \end{equation*}

where *K* is the number of components obtained by the counts of stable states in the deterministic dynamics $\mathop {}\!\mathrm{d}\boldsymbol{x}_t/\mathop {}\!\mathrm{d}t = \boldsymbol{b}(\boldsymbol{x}_t)$, $p_{(k)}(\boldsymbol{x}; \boldsymbol{\mu }_{(k)}, \boldsymbol{\Sigma }_{(k)})$ is the *k*th Gaussian component with mean $\boldsymbol{\mu }_{(k)}$ and covariance $\epsilon \boldsymbol{\Sigma }_{(k)}$, and ρ^(*k*)^ is the mixture weight. These approximations are obtained through asymptotics of (3) with respect to the small parameter ε.

*Short time asymptotics.* We denote by Ω_*k*_ the *k*th attractive basin for the $\boldsymbol{X}_t$ dynamics in (3). In the *O*(1) timescale (or the transition timescale in Ω_*k*_), there is no state hopping and we derive the approximation $\boldsymbol{X}_t\approx \boldsymbol{\mu }^{(k)}_t+\sqrt{\epsilon }\boldsymbol{Z}^{(k)}_t$, where $\boldsymbol{Z}^{(k)}_t\sim N(0,\boldsymbol{\Sigma }^{(k)}_t)$ when the initial $\boldsymbol{Y}_0\in \Omega _k$. The mean and covariance satisfy the equations

(12a) \begin{equation*} \frac{\mathop {}\!\mathrm{d}\boldsymbol{\mu }^{(k)}_t}{\mathop {}\!\mathrm{d}t} = L_1\left(\boldsymbol{\mu }^{(k)}_t, \boldsymbol{\Sigma }^{(k)}_t, \epsilon \right), \end{equation*}

(12b) \begin{equation*} \frac{\mathop {}\!\mathrm{d}\boldsymbol{\Sigma }^{(k)}_t}{\mathop {}\!\mathrm{d}t} = L_2\left(\boldsymbol{\mu }^{(k)}_t, \boldsymbol{\Sigma }^{(k)}_t, \epsilon \right), \end{equation*}

where *L*_1_ and *L*_2_ are functions derived from the Taylor expansion of $\boldsymbol{b}(\boldsymbol{x})$ around $\boldsymbol{\mu }^{(k)}_t$ until *O*(ε) terms. The details are shown in the online supplementary material. The parameters $(\boldsymbol{\mu }_{(k)}, \boldsymbol{\Sigma }_{(k)})$ are steady states of (12). Especially, $L_1(\boldsymbol{\mu },\boldsymbol{\Sigma },\epsilon )=\boldsymbol{b}(\boldsymbol{\mu })$ in the *O*(1) approximation (i.e. ε = 0) and we recover the classical MFA [17,29].

*Long time asymptotics.* The determination of the mixture weights {ρ^(*k*)^} is designed to take into account the basin transitions in a longer time scale like log (*t*) ≳ *O*(1/ε). In this regime, the diffusion dynamics of $\boldsymbol{X}_t$ is upscaled to a continuous-time Markov chain, in which the Arrhenius-type transition rates depend on the energy barriers between the corresponding attraction basins [30,31]. We assume that the upscaled transition rate matrix is $\boldsymbol{Q}$ and define $R_k = \int R(\boldsymbol{x})p_{(k)}(\boldsymbol{x}, \boldsymbol{\mu }_{(k)}, \Sigma _{(k)})\mathop {}\!\mathrm{d}\boldsymbol{x}$ as the average BDR. The evolution of weights $\boldsymbol{\rho }$ with/without a birth-death term $R(\boldsymbol{x})$ shows the asymptotics

(13) \begin{equation*} \frac{\mathop {}\!\mathrm{d}\boldsymbol{\rho }_{U}}{\mathop {}\!\mathrm{d}t} = \boldsymbol{Q}^T\boldsymbol{\rho }_U + \boldsymbol{R} \boldsymbol{\rho }_{U},\qquad \frac{\mathop {}\!\mathrm{d}\boldsymbol{\rho }_{0}}{\mathop {}\!\mathrm{d}t} = \boldsymbol{Q}^T\boldsymbol{\rho }_{0}, \end{equation*}

where $\boldsymbol{R}={\rm diag}(R_k)$, $\boldsymbol{\rho }_U=(\rho ^{(k)}_U)$ is the mixture weights for $P_U(\boldsymbol{x})$ and $\boldsymbol{\rho }_0 = (\rho ^{(k)}_0)$ is the mixture weights for $P_0(\boldsymbol{x})$. As accurate $\boldsymbol{Q}$ is difficult to obtain, we perform a Monte Carlo approximation to steady $\boldsymbol{\rho }_{0}$ by utilizing the equation

(14) \begin{equation*} \rho _{0}^{(k)} \approx \lim _{t\rightarrow +\infty }\mathbb {E}[\delta (\boldsymbol{X}_t(\omega )\in \Omega _k)], \end{equation*}

where the trajectories are simulated with a uniform initial distribution on a finite domain at *t* = 0 until a suitable finite ending time *t* = *T*. Such a choice actually gives the same MFA as proposed in [17,18] without the $R(\boldsymbol{x})$ term. With ergodic assumption, the steady mixture weights $\rho _U^{(k)}$ for $P_U(\boldsymbol{x})$ are derived as

(15) \begin{eqnarray*} \rho _U^{(k)}&\approx &\lim _{t\rightarrow +\infty }\mathbb {E}[\rho _t(\boldsymbol{X}_t(\omega ))\delta (\boldsymbol{X}_t(\omega )\in \Omega _k)]\nonumber\\ & =& \frac{\rho _0^{(k)}q_k}{q_k-R_k}, \end{eqnarray*}

where *q*_*k*_ = −*Q*_*kk*_ is the exit rate for state *k*. Under the constraint equation, (2), and an additional assumption that $1/q_k\propto \rho _0^{(k)}$, an approximation for *q*_*k*_ and thus $\rho _U^{(k)}$ is also obtained (see the online supplementary material for details).

With the mean-field approximations $P_0(\boldsymbol{x})$ and $P_U(\boldsymbol{x})$, we obtain the estimations for the landscapes $U(\boldsymbol{x})$ and $V(\boldsymbol{x})$ using (5) and (6), respectively.

Several detailed remarks need to be made. (i) There is also an MFA to (1), but, for multi-potential systems, the MFA to (1) can have different numbers of components with different parameters when approximating $P_0(\boldsymbol{x})$ and $P_U(\boldsymbol{x})$, respectively. That can lead to an odd $V(\boldsymbol{x})$. Thus, one of the advantages of the MFA to (3) is that the numbers of components *K* and $p_{(k)}(\boldsymbol{x}, \boldsymbol{\mu }_{(k)}, \Sigma _{(k)})$ computed using (12) are independent of the BDR $R(\boldsymbol{x})$, and only mixture weights estimated in (14) and (15) are different. (ii) The MFA to (3) is not suitable for monostable systems, as the mixture weight for trajectories in the only attractive basin is supposed to be the same. For the system with a uni-component, we use the MFA to (3), which is also described in detail in the online supplementary material. For real problems, most systems are multi-stable with several different components. (iii) In (12), the MFA is expanded to *O*(ε) in functions *L*_1_ and *L*_2_. In Wang’s framework [17,18] where $R(\boldsymbol{x}) \equiv 0$, it is only expanded to *O*(1) to estimate energy landscape $U(\boldsymbol{x})$. In the online supplementary material, we state that it is necessary to have the MFA to *O*(ε) to obtain a proper approximation of $V(\boldsymbol{x})$. Two analytic examples are also used to validate the necessity.

### Numerical examples

In this section, we utilize three examples to show how we applied the ELD framework to describe cell differentiation.

#### Example 1: two-dimensional drift-diffusion process

For the first example, we use a two-dimensional drift-diffusion process as a basic example for simulating cell differentiation [13,16]. In this example, we take $\boldsymbol{b}(\boldsymbol{x}) = -\nabla F(\boldsymbol{x})$ to be a gradient field with

**Figure 2.**
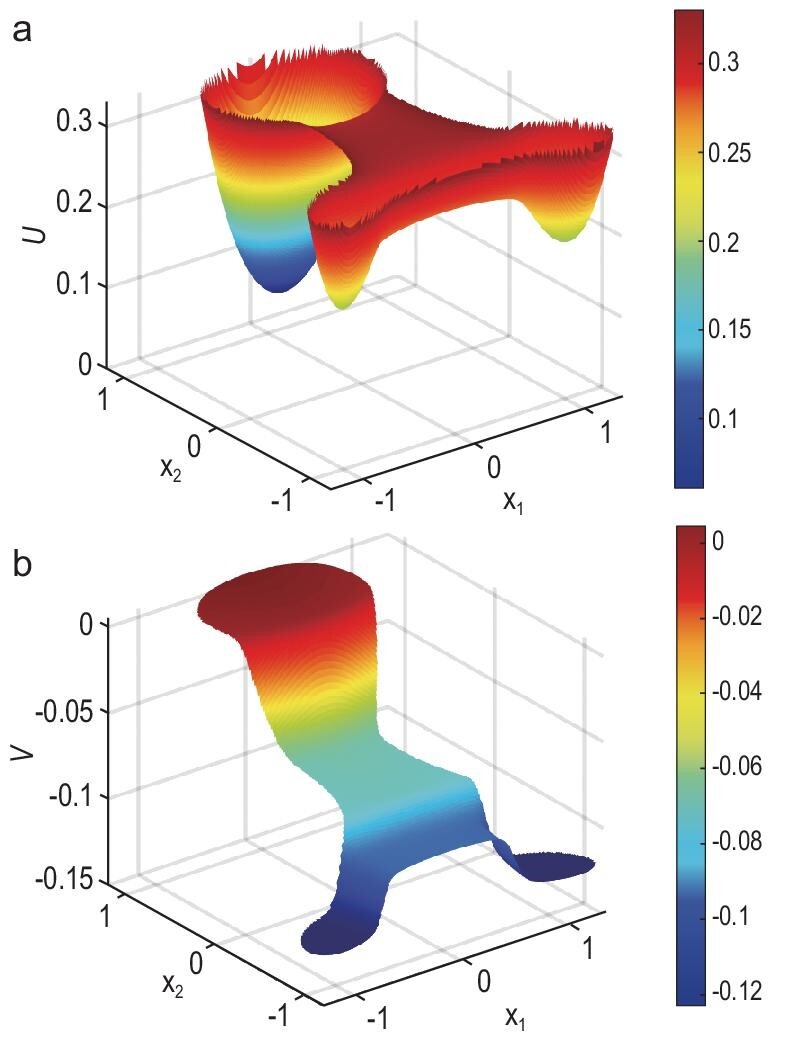
The landscapes for the two-dimensional drift-diffusion process. (a) The landscape *U*. Three metastable states represent three cell types. (b) The landscape *V*. The cells differentiate from the state at a high value of *V* to a lower value.

(16) \begin{equation*} F(\boldsymbol{x}) = \bigg (\frac{x_1^2}{2}+\frac{x_2}{2}\bigg )^2+\frac{(x_2^2-1)^2}{2}, \end{equation*}

and set the rate function $R(\boldsymbol{x}) = 0.3[(x_1^2-1)^2+(x_2+1)^2-1]$, where $\boldsymbol{x} = (x_1, x_2)^T$. The noise parameter is ε = 0.01. There exist three stable equilibrium points of the potential $F(\boldsymbol{x})$, i.e. $\boldsymbol{x}_A = (0, \sqrt{3}/2)^T$, $\boldsymbol{x}_B = (-1, -1)^T$ and $\boldsymbol{x}_C = (1, -1)^T$, which correspond to three cell types. According to $R(\boldsymbol{x})$, most cells proliferate around $\boldsymbol{x}_A$, and die around $\boldsymbol{x}_B$ and $\boldsymbol{x}_C$. Thus, cell type *A* around $\boldsymbol{x}_A$ stands for stem cells with high pluripotency, while cell types *B* around $\boldsymbol{x}_B$ and *C* around $\boldsymbol{x}_C$ represent differentiated states. Using the streamline diffusion method to solve (1) with and without $R(\boldsymbol{x})$, we obtain the distributions *P*_*U*_ and *P*_0_, respectively. Furthermore, the energy landscapes *U* and *V* are constructed as shown in Fig. 2. Three cell types are easily identified in $U(\boldsymbol{x})$. The effect of the birth and death rate $R(\boldsymbol{x})$ determines the differentiation direction of the process, and the values of $V(\boldsymbol{x})$ characterize the stemness from high to low. Figure S1 within online supplementary material also shows the original potential $F(\boldsymbol{x})$, together with the two-dimensional projections of *U* and *V*.

#### Example 2: the two-gene fate decision system

**Figure 3.**
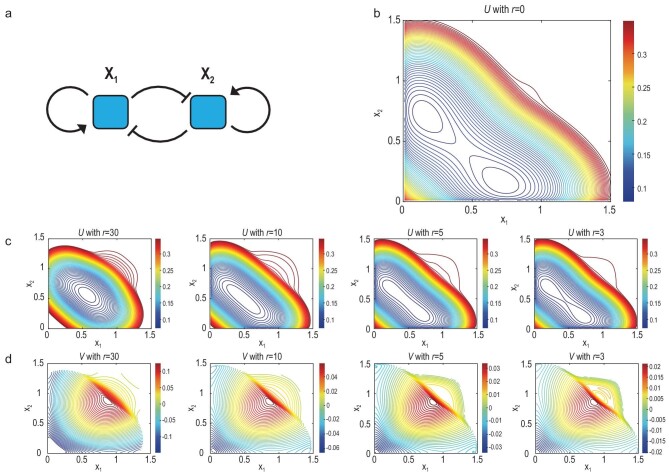
(a) The interaction between two genes *X*_1_ and *X*_2_ in the fate decision system. Self-activation and inter-inhibition are observed. (b) The landscape *U* when the amplitude of the birth and death rate is set as *r* = 0. (c) The landscape *U* changes when *r* decreases from 30 to 3. (d) The landscape *V* changes when *r* decreases from 30 to 3.

The second example models a binary cell fate decision controlled by the interaction between two genes [18,32,33]. Figure 3a shows the regulatory relationships between the two genes. It is modeled using a non-gradient Hill dynamics as

(17) \begin{equation*} \boldsymbol{b}(\boldsymbol{x}) = \left[{\begin{array}{c}\frac{\alpha _1 x_1^n}{S^n+x_1^n} + \frac{\beta _1S^n}{S^n+x_2^n} - k_1x_1 \\ \frac{\alpha _2x_2^n}{S^n+x_2^n}+\frac{\beta _2S^n}{S^n+x_1^n} - k_2x_2 \end{array}}\right], \end{equation*}

where the parameters are set as α_1_ = α_2_ = 0.3, β_1_ = β_2_ = 0.5, *n* = 4, *k*_1_ = *k*_2_ = 1 and *S* = 0.5. Through self-activation and inter-inhibition, the two genes *X*_1_ and *X*_2_ (such as GATA1 and PU.1) are coexpressed in the pluripotent stem cell, and one gene gradually dominates over the other when the system is committed to two different lineages. In Wang’s framework, α_1_, α_2_, β_1_ and β_2_ vary to control the differentiation [18]. However, in the current viewpoint of cell differentiation, ELD theory claims that, even if these parameters are fixed, the cell fate decision can be adjusted according to the change in BDR $R(\boldsymbol{x})$. To construct the landscapes *U* and *V*, we set the noise amplitude ε = 0.01, and the birth and death rate $R(\boldsymbol{x}) = -r[(x_1-1)^2+(x_2-1)^2-0.5]$. The rate amplitude *r* is changed from 30 to 0. Figure 3c and d demonstrate how the landscapes change when *r* decreases, and Fig. 3b shows the landscape of *U* when *r* = 0 (the landscape *V* ≡ 0). We obtain the following results through our computations: (i) when the BDR is high (*r* = 30), there is only one cell type characterized by *U*, where *X*_1_ and *X*_2_ are coexpressed; (ii) this single state splits into two as the amplitude of BDR decreases, i.e. differentiation; (iii) in the two separated cell types when *r* = 0, one gene dominates over the other; (iv) the value of *V* characterizes the pluripotency of cells, and −∇*V* indicates the differentiation direction. Overall, instead of changing the interaction strength between genes, the BDR term $R(\boldsymbol{x})$ might also be responsible for explaining cell differentiation, which controls the cell fate decision.

#### Example 3: T-cell differentiation

**Figure 4.**
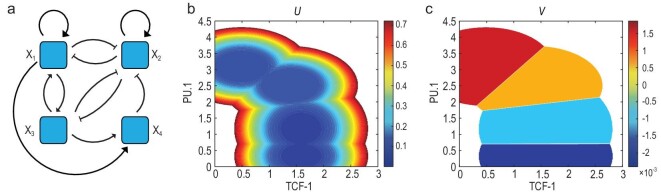
(a) The gene regulatory network in the T-cell differentiation process. The four genes *X*_1_, *X*_2_, *X*_3_ and *X*_4_ are TCF-1, PU.1, GATA3 and BLC11B, respectively. (b) The landscape *U* and (c) the landscape *V*. Metastable states represent the T-cell stages, i.e. ETP/DN1, DN2a, DN2b and DN3. The potential *V* indicates the pluripotency and differentiation direction.

The third example is used to describe the T-cell differentiation in high-dimensional space [34]. Four genes *x*_1_ (TCF-1), *x*_2_ (PU.1), *x*_3_ (GATA3) and *x*_4_ (BLC11B) interact with each other through activation or inhibition (see Fig. 4a). The dynamical term $\boldsymbol{b}(\boldsymbol{x})$ is modeled using Hill functions and the parameters are listed in the online supplementary material. We set the BDR as $R(\boldsymbol{x}) = 30[4.2-x_1^2 - (x_2-4)^2]$. Using the MFA equation, (11), the landscapes are constructed as shown in Fig. 4b and c. The potential *U* corresponds to the steady-state landscape with birth-death term *R*. The four metastable states stand for the four stages of development of T cells (ETP/DN1, DN2a, DN2b and DN3). The potential *V* in Fig. 4c shows the pluripotency of cells with its value and differentiation direction with its negative gradient field. As applied to MFA, the BDR is also averaged for each Gaussian component, so the variation of $V(\boldsymbol{x})$ within each metastable state/cell type is much smaller than that shown by *U*. Accordingly, there exists a sharp variation of *V* between two adjacent cell types, as shown in Fig. 4c.

Besides the four-dimensional example for T-cell differentiation, we also conducted ELD on a high-dimensional example with 10 variables shown in Sec. IV(D) and Figure S2 of the online supplementary material. These results support the fact that the ELD and MFA are practical in studying the metastable states by energy landscape *U* and the pluripotency by energy landscape *V*.

## CONCLUSIONS AND DISCUSSIONS

In this paper, we have proposed the ELD framework to describe cell differentiation with proliferation effect. Two energy landscapes, $U(\boldsymbol{x})$ and $V(\boldsymbol{x})$, can explain the dynamical behavior of the system during differentiation. The potential *U* depicts the attractors standing for cell types, while *V* characterizes the pluripotency and differentiation direction. The consideration of BDR is important to construct *V*. With an additional *V*, ELD theory is a generalization of traditional energy landscape theory, and it is a natural realization for Waddington’s epigenetic landscape with birth-death terms. The numerical construction of ELD, especially its mean-field approximation in high-dimensional cases, is introduced. Simulated examples demonstrate the applicability of ELD. However, there are still several issues that need to be further discussed and studied.

First, the ELD framework is different from other models for constructing landscapes for cell differentiation. PBA and LDD are data driven, which are based on the scRNA-seq data and difficult to handle the non-gradient system. Wang’s landscape does not consider the birth and death of cells during the differentiation. ELD is model based and analyzes a landscape *V* caused by cell proliferation and death to display the differentiation direction.

Second, the BDR $R(\boldsymbol{x})$ in our model is known a priori. For practical cases, *R* can be estimated from experiments (as in PBA, [13]) or approximated for each cell type (as in LDD, [16]). If only *R* for cell types matters, which means that *R* is a constant for each metastable state, the corresponding *V* will change little within one state while varying sharply between two adjacent states.

Third, the estimated mixture weights $\rho _0^{(k)}$ in (14) are only rough approximations according to the size of attractive basins (percentages of trajectories falling into one meta-stable state). A more rational approach is to get the transition rate matrix $\boldsymbol{Q}$ between different states [31,35–37]. However, it is difficult to catch rare transitions within the limited time of the simulation. A quick and accurate way to estimate mixture weights is still an open question.

Fourth, constructing landscapes for systems with limit cycles is also possible once a proper BDR is given. According to the approximation in [17], $p_{(k)}(\boldsymbol{x}) = \lim _{s\rightarrow +\infty }(1/T)\int _s^{s+T} p(\boldsymbol{x}, \boldsymbol{\mu }_t, \Sigma _t)\mathop {}\!\mathrm{d}t$ can be used as the *k*th component in (11), where *T* is the period for the limit cycle and $p(\boldsymbol{x}; \boldsymbol{\mu }_t, \Sigma _t)$ is the Gaussian mean-field approximation of the PDF at *t* by simulating (12). Constructing landscapes for real systems with limit-cycle behavior will be our future work.

Finally, in this paper we used FPE with a BDR term to study the dynamical behavior of the differentiation process in normal cells. For cancer cells that developed from normal cells, the pluripotency and proliferation rates may be restored by external factors. We leave the study of the energy landscape for cancer or tumor cells to future work.

Overall, the energy landscape is a universal concept to characterize the dynamical behavior of a system, and the proposed ELD in this study can help understand systems with proliferation and death, beyond pure reactions. This work can also be applied to model-based and data-based dynamical analyses of various biological systems [38–42].

## MATERIALS AND METHODS

Detailed methods are available in the online supplementary material.

## Supplementary Material

nwac116_Supplemental_File
