# Supplementary material for: Energy landscape decomposition for cell differentiation with proliferation effect
Source: Natl Sci Rev. 2022 Jun 17;9(8):nwac116. doi: 10.1093/nsr/nwac116 (PMC9385468; doi:10.1093/nsr/nwac116)
Supplement: nwac116_Supplemental_File [file nwac116_supplemental_file.pdf]

Supplementary Information for "Energy landscape decomposition for cell differentiation with proliferation effect"

Jifan Shi,[1,2,*] Kazuyuki Aihara,[2,†] Tiejun Li,[3,‡] and Luonan Chen[4,5,6,7,§]

[1] *Research Institute of Intelligent Complex Systems, Fudan University, Shanghai 200433, China*
[2] *International Research Center for Neurointelligence, The University of Tokyo Institutes for Advanced Study, The University of Tokyo, Tokyo 113-0033, Japan*
[3] *LMAM and School of Mathematical Sciences, Peking University, Beijing 100871, China*
[4] *Key Laboratory of Systems Biology, Shanghai Institute of Biochemistry and Cell Biology, Center for Excellence in Molecular Cell Science, Chinese Academy of Sciences, Shanghai 200031, China*
[5] *Key Laboratory of Systems Health Science of Zhejiang Province, Hangzhou Institute for Advanced Study, University of Chinese Academy of Sciences, Chinese Academy of Sciences, Hangzhou 310024, China*
[6] *School of Life Science and Technology, ShanghaiTech University, Shanghai 201210, China*
[7] *Guangdong Institute of Intelligence Science and Technology, Zhuhai 519031, China*

Contents

I. Theory of the energy landscape decomposition 3
A. Gradient system 3
B. No birth and death system 4
C. Weighted stochastic equation form 4

II. Numerical algorithm for the low dimensional system 5

III. Mean-field approximation in the high dimensional case 6
A. MFA for the weighted dynamical system 7
1. Short time asymptotics 7
2. Long time asymptotics 8
B. MFA for the population-level equation 10
C. Analytic solution of the mean-field approximation for two examples 11
1. 1-dim gradient system 11
2. 2-dim non-gradient system 11

IV. Details of the numerical examples 12
A. Drift-diffusion process 12

*Electronic address: shijifan@ircn.jp
†Electronic address: kaihara@g.ecc.u-tokyo.ac.jp
‡Electronic address: tieli@pku.edu.cn
§Electronic address: lnchen@sibs.ac.cn

B. Two-gene fate decision 12
C. T-cell differentiation process 13
D. 10-dimensional system with the birth and death term 13

References 14

I. THEORY OF THE ENERGY LANDSCAPE DECOMPOSITION

We begin with the cell population equation

$$\frac{\partial p(\boldsymbol{x},t)}{\partial t} = -\nabla \cdot (\boldsymbol{b}(\boldsymbol{x})p(\boldsymbol{x},t)) + \epsilon\Delta p(\boldsymbol{x},t) + R(\boldsymbol{x})p(\boldsymbol{x},t), \tag{S1}$$

where $\boldsymbol{x} \in \mathbb{R}^n$ is the gene expression vector, $\boldsymbol{b}(\boldsymbol{x})$ is the drift term, ϵ is the noise amplitude, $R(\boldsymbol{x})$ is the birth and death rate (BDR), and $p(\boldsymbol{x},t)$ is the probability density function (pdf) of cells over the gene expression space.

The energy landscape decomposition (ELD) is to find energy landscapes $U(\boldsymbol{x})$, $V(\boldsymbol{x})$, and curl part term $\boldsymbol{f}(\boldsymbol{x})$ which satisfy

$$\boldsymbol{b}(\boldsymbol{x}) = -\nabla U(\boldsymbol{x}) - \nabla V(\boldsymbol{x}) + \boldsymbol{f}(\boldsymbol{x}). \tag{S2}$$

The decomposition has infinite choices, but a meaningful one is supposed to use U to describe the cell types and use V to describe the differentiation. There are data-based approaches and model-based approaches for constructing energy landscape. In this study, ELD is a model-based approach with assumption that $\boldsymbol{b}(\boldsymbol{x}), \epsilon$ and $R(\boldsymbol{x})$ are known, which means we can solve Eq. S1 or simulate a corresponding stochastic system Eq. S10 to get the final steady pdf, denoted as $P_U(\boldsymbol{x})$. Thus, U is defined by

$$U(\boldsymbol{x}) = -\epsilon \log P_U(\boldsymbol{x}). \tag{S3}$$

U can be taken as the simplest gradient system whose equilibrium density equals to $P_U(\boldsymbol{x})$. Metastable states on U stand for different cell types. On the other hand, by setting $R(\boldsymbol{x})$ as zero, we can obtain another steady pdf denoted as $P_0(\boldsymbol{x})$. V is defined by

$$V(\boldsymbol{x}) = -\epsilon \log \frac{P_0(\boldsymbol{x})}{P_U(\boldsymbol{x})}. \tag{S4}$$

V can be taken as the change of landscape U caused by the cell birth and death. Values of V can represent the pluripotency, and the differentiation evolves from the high to the low value of V. The remained part in $\boldsymbol{b}(\boldsymbol{x})$ is the curl part term defined as

$$\boldsymbol{f}(\boldsymbol{x}) = \boldsymbol{b}(\boldsymbol{x}) + \nabla U(\boldsymbol{x}) + \nabla V(\boldsymbol{x}) = \boldsymbol{b}(\boldsymbol{x}) - \epsilon\nabla \log P_0(\boldsymbol{x}). \tag{S5}$$

$\boldsymbol{f}$ satisfies the property $\nabla \cdot (\boldsymbol{f}(\boldsymbol{x})P_0(\boldsymbol{x})) = 0$, which corresponds to the divergence free condition of the curl flux $\boldsymbol{J}(\boldsymbol{x}) = \boldsymbol{f}(\boldsymbol{x})P_0(\boldsymbol{x})$ defined in [1, 2].

A. Gradient system

A special case of Eq. S1 is $\boldsymbol{b}(\boldsymbol{x}) = -\nabla F(\boldsymbol{x})$, which is a gradient system. We can obtain the analytic expression of $P_0(\boldsymbol{x})$ as

$$P_0(\boldsymbol{x}) = \frac{1}{Z_0}\exp(-\frac{F(\boldsymbol{x})}{\epsilon}), \tag{S6}$$

where Z_0 is a normalization constant. By Eq. S3, Eq. S4, Eq. S5, and Eq. S6, we can obtain $F(\boldsymbol{x}) = U(\boldsymbol{x}) + V(\boldsymbol{x})$, in the sense of ignoring a constant, and $\boldsymbol{f}(\boldsymbol{x}) = \boldsymbol{0}$. Further, through the Eq. S3 and the Eq. S1 at $t = +\infty$, we can obtain that the potential U satisfies a forward equation

$$\mathcal{L}_U^* U = \nabla \cdot (\nabla U(\boldsymbol{x})P_U(\boldsymbol{x})) + \epsilon\Delta P_U(\boldsymbol{x}) = 0, \tag{S7}$$

and the potential V satisfies another backward equation

$$\mathcal{L}_V V = \nabla \log P_U(\boldsymbol{x}) \cdot \nabla V(\boldsymbol{x}) + \Delta V(\boldsymbol{x}) = -R(\boldsymbol{x}). \tag{S8}$$

Thus, for a gradient system, the decomposition Eq. S2 degenerates to the methods used in the population balance analysis (PBA) [3] and the landscape of differentiation dynamics (LDD) [4]. The difference is that PBA and LDD are data-based, and estimate U and V by solving Eq. S7 and Eq. S8 from real datasets. The EDL in this paper is model-based and tries to estimate U and V by Eq. S3 and Eq. S4.

As a remark, for a non-gradient system, Eq. S7 always holds, while Eq. S8 doesn't. Through

$$\begin{aligned} R(\boldsymbol{x}) &= \frac{1}{P_U(\boldsymbol{x})} \cdot [\nabla \cdot (\boldsymbol{b}(\boldsymbol{x}) P_U(\boldsymbol{x})) - \epsilon \Delta P_U(\boldsymbol{x})] = -\frac{1}{P_U(\boldsymbol{x})} \nabla \cdot [(\nabla V(\boldsymbol{x}) - \boldsymbol{f}(\boldsymbol{x})) P_U(\boldsymbol{x})] \\ &= -\nabla \log P_U(\boldsymbol{x}) \cdot \nabla V(\boldsymbol{x}) - \Delta V(\boldsymbol{x}) + \boldsymbol{f} \cdot \log \frac{P_U(\boldsymbol{x})}{P_0(\boldsymbol{x})} \\ &= -\nabla \log P_U(\boldsymbol{x}) \cdot \nabla V(\boldsymbol{x}) - \Delta V(\boldsymbol{x}) + \frac{1}{\epsilon} \boldsymbol{f}(\boldsymbol{x}) \cdot \nabla V(\boldsymbol{x}), \end{aligned} \tag{S9}$$

we know Eq. S8 holds if and only if $\boldsymbol{f} \perp \nabla V$. Estimating V by solving Eq. S8 in PBA and LDD can not be generalized to the non-gradient system.

B. No birth and death system

When the system has no birth and death, i.e. $R(\boldsymbol{x}) \equiv 0$, we can obtain $P_0(\boldsymbol{x}) \equiv P_U(\boldsymbol{x})$ and $V(\boldsymbol{x}) \equiv 0$. Thus the decomposition Eq. S2 reduces to the landscape proposed by Wang et al. [1, 2]. But Wang's landscape is constructed for each set of possible parameters, such as different strength of gene-gene interaction or cell volume. The change of the landscape U during the differentiation is controlled by the manually setting parameters. However, the parameters in our ELD can be fixed. If the essential proliferation and death of cells are taken into account, i.e. $R(\boldsymbol{x}) \not\equiv 0$, we show that an induced landscape V can characterize the direction of differentiation.

C. Weighted stochastic equation form

There exists a weighted stochastic dynamics corresponding to the population equation Eq. S1 as

$$\begin{cases} \mathrm{d}\boldsymbol{X}_t(\boldsymbol{y},\omega) &= \boldsymbol{b}(\boldsymbol{X}_t(\boldsymbol{y},\omega))\,\mathrm{d}t + \sqrt{2\epsilon} \cdot \mathrm{d}\boldsymbol{W}_t(\boldsymbol{y},\omega), \\ \boldsymbol{X}_{t=0}(\boldsymbol{y},\omega) &= \boldsymbol{y}, \\ \mathrm{d}\rho_t(\boldsymbol{y},\omega) &= R(\boldsymbol{X}_t(\boldsymbol{y},\omega))\rho_t(\boldsymbol{y},\omega)\,\mathrm{d}t, \\ \rho_{t=0}(\boldsymbol{y},\omega) &= 1, \end{cases} \tag{S10}$$

where $\boldsymbol{X}_t(\boldsymbol{y},\omega)$ is a trajectory ω with the initial point $\boldsymbol{y}$, and $\rho_t(\boldsymbol{y},\omega)$ is the weight for this trajectory. Each trajectory represents a cell in the differentiation process. All the trajectories have the same weight as $t = 0$. If the weight ρ of ω increases, it is supposed that the cell has proliferation, while if the weight ρ of ω decreases, death is supposed to occur. The population pdf is computed as the weighted expectation of all trajectories, i.e. $p(\boldsymbol{x},t) = \mathbb{E}\left\{\rho_t(\boldsymbol{y},\omega)\delta(\boldsymbol{x} - \boldsymbol{X}_t(\boldsymbol{y},\omega))\right\}$, where $\delta(\cdot)$ is the Dirac delta function.

Proposition: The population pdf of stochastic system Eq. S10 satisfies Eq. S1.
Proof: By Ito formula, we have

$$\begin{aligned}\frac{\partial p(\boldsymbol{x},t)}{\partial t} &= \frac{\mathrm{d}}{\mathrm{d}t}\mathbb{E}_{(\boldsymbol{y},\omega)}\Big\{\rho_t(\boldsymbol{y},\omega)\delta(\boldsymbol{x}-\boldsymbol{X}_t(\boldsymbol{y},\omega))\Big\}\\ &= \frac{1}{\mathrm{d}t}\mathbb{E}_{(\boldsymbol{y},\omega)}\Big\{\mathrm{d}\rho_t(\boldsymbol{y},\omega)\cdot\delta(\boldsymbol{x}-\boldsymbol{X}_t(\boldsymbol{y},\omega))+\rho_t(\boldsymbol{y},\omega)\big[\delta'(\boldsymbol{x}-\boldsymbol{X}_t(\boldsymbol{y},\omega))\cdot(-\mathrm{d}\boldsymbol{X}_t)+\frac{1}{2}\delta''(\boldsymbol{x}-\boldsymbol{X}_t)\cdot(\mathrm{d}\boldsymbol{X}_t)^2\big]\Big\}\\ &= \frac{1}{\mathrm{d}t}\mathbb{E}_{(\boldsymbol{y},\omega)}\Big\{R(\boldsymbol{X}_t(\boldsymbol{y},\omega))\rho_t\delta(\boldsymbol{x}-\boldsymbol{X}_t)\,\mathrm{d}t-\rho_t\delta'(\boldsymbol{x}-\boldsymbol{X}_t)\cdot(b(\boldsymbol{X}_t)\,\mathrm{d}t+\sqrt{2\epsilon}\,\mathrm{d}\boldsymbol{W}_t)+\frac{1}{2}\rho_t\delta''(\boldsymbol{x}-\boldsymbol{X}_t)\cdot 2\epsilon\,\mathrm{d}t\Big\}\\ &= \mathbb{E}_{(\boldsymbol{y},\omega)}\Big\{R(\boldsymbol{x})\rho_t\delta(\boldsymbol{x}-\boldsymbol{X}_t)-\partial_x\big(\rho_t\delta(\boldsymbol{x}-\boldsymbol{X}_t)\cdot b(\boldsymbol{x})\big)+\epsilon\partial_{xx}\big(\rho_t\delta(\boldsymbol{x}-\boldsymbol{X}_t)\big)\Big\}\\ &= R(\boldsymbol{x})p(\boldsymbol{x},t)-\nabla\cdot(\boldsymbol{b}(\boldsymbol{x})p(\boldsymbol{x},t))+\epsilon\Delta p(\boldsymbol{x},t),\end{aligned} \tag{S11}$$

which is the same as Eq. S1. The initial $p(\boldsymbol{x},0)$ is set as the distribution of $\boldsymbol{y}$. □

II. NUMERICAL ALGORITHM FOR THE LOW DIMENSIONAL SYSTEM

The key point to obtain the decomposition is to estimate the two steady pdfs $P_U(\boldsymbol{x})$ and $P_0(\boldsymbol{x})$. For the low-dimensional system, we can use numerical algorithms, such as finite difference or finite element method, to solve Eq. S1. In the following, we will show the details of streamline diffusion method [5], which is applied in our examples.

The weak form of Eq. S1 in streamline diffusion method is

$$\langle\frac{\partial p}{\partial t},v+\delta v_{\boldsymbol{b}}\rangle=-\epsilon\langle\nabla p,\nabla v\rangle+\epsilon\delta\langle\Delta p,v_{\boldsymbol{b}}\rangle-\langle\nabla\cdot(\boldsymbol{b}p),v+\delta v_{\boldsymbol{b}}\rangle+\langle Rp,v+\delta v_{\boldsymbol{b}}\rangle, \tag{S12}$$

where $\langle\cdot,\cdot\rangle$ is the integration over the considered spatial space Ω, $v+\delta v_{\boldsymbol{b}}$ is the test function, $v\in\mathcal{H}_0^1(\Omega)$, $v_{\boldsymbol{b}}=\boldsymbol{b}\cdot\nabla v$, and δ is a small constant.

In our 2-dim examples, we use the bilinear function over rectangle grid in the spatial axis. On each grid element, we assume $\boldsymbol{b}(\boldsymbol{x}),\nabla\cdot\boldsymbol{b}(\boldsymbol{x}),R(\boldsymbol{x})$ are constants with the value at the left-bottom point. If we choose the implicit 1-order discretization of the temporal axis and denote the basis over spatial grid as $\{\varphi_i(\boldsymbol{x})\}$, the Eq. S12 is discretized as

$$\begin{aligned}\sum_i\Big\{\langle\varphi_j,\varphi_i\rangle+\delta\langle\boldsymbol{b}\cdot\nabla\varphi_j,\varphi_i\rangle-\tau\big[-\epsilon\langle\nabla\varphi_j,\nabla\varphi_i\rangle+\epsilon\delta\langle\boldsymbol{b}\cdot\nabla\varphi_j,\Delta\varphi_i\rangle-\langle\varphi_j+\delta(\boldsymbol{b}\cdot\nabla\varphi_j),(\nabla\cdot\boldsymbol{b})\cdot\varphi_i+\boldsymbol{b}\cdot\nabla\varphi_i-R\varphi_i\rangle\big]\Big\}p_i^{t+\tau}\\ =\sum_i\Big\{\langle\varphi_j,\varphi_i\rangle+\delta\langle\boldsymbol{b}\cdot\nabla\varphi_j,\varphi_i\rangle\Big\}p_i^t,\end{aligned} \tag{S13}$$

where τ is the time step, and p_i^t is the value of $p(\boldsymbol{x},t)$ at the grid point $\boldsymbol{x}_i$. Eq. S13 is a linear equation, which can be denoted as

$$A\boldsymbol{p}^{t+\tau}=B\boldsymbol{p}^t, \tag{S14}$$

where $A\in\mathbb{R}^{N\times N}$, $B\in\mathbb{R}^{N\times N}$, $\boldsymbol{p}^t\in\mathbb{R}^{N\times 1}$, and N is the number of grid nodes in Ω. For each square finite element K with spatial step size h, the bilinear bases are

$$\begin{aligned}\varphi^{(1)}(\boldsymbol{x})&=\left(1-\frac{x_1-a}{h}\right)\left(1-\frac{x_2-b}{h}\right), & \varphi^{(2)}(\boldsymbol{x})&=\frac{x_1-a}{h}\left(1-\frac{x_2-b}{h}\right),\\ \varphi^{(3)}(\boldsymbol{x})&=\frac{x_1-a}{h}\frac{x_2-b}{h}, & \varphi^{(4)}(\boldsymbol{x})&=\left(1-\frac{x_1-a}{h}\right)\frac{x_2-b}{h},\end{aligned} \tag{S15}$$

where $\boldsymbol{x}_K=(a,b)$ is the left-bottom point of K. With Dirichlet boundary condition, the global A and B can be assembled by the local matrices

$$\begin{aligned}A_K=&\frac{\tau\cdot\epsilon}{6}M_1+h^2\cdot\frac{1+(\nabla\cdot\boldsymbol{b}_K-R_K)\tau}{36}M_2+h\cdot\frac{\delta+\delta(\nabla\cdot\boldsymbol{b}_K-R_K)\tau}{12}\cdot b_K^{(1)}M_3+h\cdot\frac{\delta+\delta(\nabla\cdot\boldsymbol{b}_K-R_K)\tau}{12}\cdot b_K^{(2)}M_4\\ &+\frac{\tau h}{12}b_K^{(1)}M_5+\frac{\tau h}{12}b_K^{(2)}M_6+\frac{\tau\delta h}{6}b_K^{(1)}b_K^{(1)}M_7+\frac{\tau\delta h}{2}b_K^{(1)}b_K^{(2)}M_8+\frac{\tau\delta h}{6}b_K^{(2)}b_K^{(2)}M_9,\end{aligned} \tag{S16}$$

and

$$B_K = \frac{h^2}{36} M_2 + \frac{\delta h}{12} b_K^{(1)} M_3 + \frac{\delta h}{12} b_K^{(2)} M_4, \tag{S17}$$

where

$$M_1 = \begin{bmatrix} 4 & -1 & -2 & -1 \\ -1 & 4 & -1 & -2 \\ -2 & -1 & 4 & -1 \\ -1 & -2 & -1 & 4 \end{bmatrix}, \quad M_2 = \begin{bmatrix} 4 & 2 & 1 & 2 \\ 2 & 4 & 2 & 1 \\ 1 & 2 & 4 & 2 \\ 2 & 1 & 2 & 4 \end{bmatrix}, M_3 = \begin{bmatrix} -2 & -2 & -1 & -1 \\ 2 & 2 & 1 & 1 \\ 1 & 1 & 2 & 2 \\ -1 & -1 & -2 & -2 \end{bmatrix},$$
$$M_4 = \begin{bmatrix} -2 & -1 & -1 & -2 \\ -1 & -2 & -2 & -1 \\ 1 & 2 & 2 & 1 \\ 2 & 1 & 1 & 2 \end{bmatrix}, \quad M_5 = \begin{bmatrix} -2 & 2 & 1 & -1 \\ -2 & 2 & 1 & -1 \\ -1 & 1 & 2 & -2 \\ -1 & 1 & 2 & -2 \end{bmatrix}, M_6 = \begin{bmatrix} -2 & -1 & 1 & 2 \\ -1 & -2 & 2 & 1 \\ -1 & -2 & 2 & 1 \\ -2 & -1 & 1 & 2 \end{bmatrix}, \tag{S18}$$
$$M_7 = \begin{bmatrix} 2 & -2 & -1 & 1 \\ -2 & 2 & 1 & -1 \\ -1 & 1 & 2 & -2 \\ 1 & -1 & -2 & 2 \end{bmatrix}, \quad M_8 = \begin{bmatrix} 1 & 0 & -1 & 0 \\ 0 & -1 & 0 & 1 \\ -1 & 0 & 1 & 0 \\ 0 & 1 & 0 & -1 \end{bmatrix}, M_9 = \begin{bmatrix} 2 & 1 & -1 & -2 \\ 1 & 2 & -2 & -1 \\ -1 & -2 & 2 & 1 \\ -2 & -1 & 1 & 2 \end{bmatrix},$$

$\boldsymbol{b}_K = (b_K^{(1)}, b_K^{(2)})^T = (b^{(1)}(\boldsymbol{x}_K), b^{(2)}(\boldsymbol{x}_K))^T$, $\nabla \cdot \boldsymbol{b}_K = \frac{b^{(1)}(a+h,b)-b^{(1)}(a,b)}{h} + \frac{b^{(2)}(a,b+h)-b^{(2)}(a,b)}{h}$, and $R_K = R(\boldsymbol{x}_K)$.

After sufficient iterations of Eq. S14 with normalized $\boldsymbol{p}$ at each time step, we can obtain an estimation of the steady distribution $\widehat{p}(\boldsymbol{x})$ on the grid.

There are two remarks: 1. The purpose of the streamline diffusion method is to reduce the oscillation caused by the numerical algorithm, especially around the shock wave domain. 2. δ is usually chosen as $\bar{c}h$, where h is the spatial grid size, $\bar{c}$ is a small positive number if $\epsilon < h$, or 0 if $\epsilon \geqslant h$.

III. MEAN-FIELD APPROXIMATION IN THE HIGH DIMENSIONAL CASE

It is not possible to solve Eq. S1 directly in the high-dimensional case. Instead, a mean-field approximation (MFA) can give a computable estimation of the steady pdfs P_U and P_0 as a Gaussian mixture form

$$\widehat{p}(\boldsymbol{x}) = \sum_{k=1}^{K} \rho^{(k)} \cdot \widehat{p}_{(k)}(\boldsymbol{x}, \boldsymbol{\mu}_{(k)}, \Sigma_{(k)}), \tag{S19}$$

where K is the number of components obtained by the counts of stable states in the deterministic dynamics $\dot{\boldsymbol{X}}_t = \boldsymbol{b}(\boldsymbol{X}_t)$,

$$\widehat{p}_{(k)}(\boldsymbol{x}, \boldsymbol{\mu}_{(k)}, \Sigma_{(k)}) = \frac{1}{(2\pi)^{\frac{N}{2}} |\epsilon \Sigma_{(k)}|^{\frac{1}{2}}} \exp\left(-\frac{1}{2\epsilon} (\boldsymbol{x} - \boldsymbol{\mu}_{(k)}^T) \Sigma_{(k)}^{-1} (\boldsymbol{x} - \boldsymbol{\mu}_{(k)}) \right), \tag{S20}$$

is the kth Gaussian component with mean $\boldsymbol{\mu}_{(k)}$ and covariance $\epsilon \Sigma_{(k)}$, and $\rho^{(k)}$ is the mixture weight with $\sum_{k=1}^{K} \rho^{(k)} = 1$.

A. MFA for the weighted dynamical system

In this subsection, we show the MFA for Eq. S10, which is also applied in our numerical example. There are two parts in the MFA Eq. S19. Gaussian component Eq. S20 is obtained by short time asymptotics, while mixture weight $\rho^{(k)}$ is obtained by long time asymptotics.

1. *Short time asymptotics*

In $O(1)$ time scale, the particle/cell stays in one attractive basin without transition. We can have the approximation

$$\boldsymbol{X}_t = \boldsymbol{\mu}_t + \sqrt{\epsilon}\boldsymbol{Z}_t, \tag{S21}$$

where $\boldsymbol{\mu}_t = \{\mu_t^i\}_{n\times 1}$ is the mean path, $\boldsymbol{Z}_t \sim \mathcal{N}(\boldsymbol{0}, \Sigma_t)$ is Gaussian random variable, and $\Sigma_t = \{\Sigma_t^{i,j}\}_{n\times n}$ is the covariance matrix of $\boldsymbol{Z}_t$. It is equivalent to approximating the pdf of cell population as $p(\boldsymbol{x},t) = \mathbb{E}_\omega\left\{\delta(\boldsymbol{x} - \boldsymbol{X}_t(\omega))\right\} \sim \mathcal{N}(\boldsymbol{\mu}_t, \epsilon\Sigma_t)$ at time t. Denote the Taylor expansion of $\boldsymbol{b}(\boldsymbol{x}) = \{b^i(\boldsymbol{x})\}_{n\times 1}$ as

$$\begin{aligned} b^i(\boldsymbol{x}) =& b^i(\boldsymbol{\mu}_t) + \nabla b^i(\boldsymbol{\mu}_t)\cdot(\boldsymbol{x}-\boldsymbol{\mu}_t) + \frac{1}{2}\nabla^2 b^i(\boldsymbol{\mu}_t) : (\boldsymbol{x}-\boldsymbol{\mu}_t)^{(2)} \\ &+ \frac{1}{3!}\nabla^3 b^i(\boldsymbol{\mu}_t) : (\boldsymbol{x}-\boldsymbol{\mu}_t)^{(3)} + \frac{1}{4!}\nabla^4 b^i(\boldsymbol{\mu}_t) : (\boldsymbol{x}-\boldsymbol{\mu}_t)^{(4)} + o(||\boldsymbol{x}-\boldsymbol{\mu}_t||^4), \end{aligned} \tag{S22}$$

where $(\boldsymbol{x}-\boldsymbol{\mu}_t)^{(k)}$ is a k-dimensional tensor whose $\boldsymbol{j} = (j_1, j_2, \ldots, j_k)$-th element is $\prod_{s=1}^{k}(x_{j_s} - \mu_{j_s})$, and the operator $A : B = \sum_{\boldsymbol{j}} A_{\boldsymbol{j}} B_{\boldsymbol{j}}$ is the summation of corresponding element-wise product of two tensors. We can find that $\boldsymbol{X}_t$ is independent of $R(\boldsymbol{x})$ in Eq. S10. Then the evolution equations for $\boldsymbol{\mu}_t$ and Σ_t are

$$\frac{\mathrm{d}\mu_t^i}{\mathrm{d}t} = b^i(\boldsymbol{\mu}_t) + \frac{\epsilon}{2}\nabla^2 b^i(\boldsymbol{\mu}_t) : \Sigma_t + O(\epsilon^2), \tag{S23}$$

and

$$\begin{aligned} \frac{\mathrm{d}\Sigma_t^{ij}}{\mathrm{d}t} =& \sum_k \partial_k b^i(\boldsymbol{\mu}_t)\Sigma_t^{kj} + \sum_k \partial_k b^j(\boldsymbol{\mu}_t)\Sigma_t^{ki} + 2\delta_{ij} \\ &+ \frac{\epsilon}{6}\sum_{l,m,s} \partial^3_{lms} b^i(\boldsymbol{\mu}_t)\cdot(\Sigma_t^{lm}\Sigma_t^{sj} + \Sigma_t^{ls}\Sigma_t^{mj} + \Sigma_t^{lj}\Sigma_t^{ms}) \\ &+ \frac{\epsilon}{6}\sum_{l,m,s} \partial^3_{lms} b^j(\boldsymbol{\mu}_t)\cdot(\Sigma_t^{lm}\Sigma_t^{si} + \Sigma_t^{ls}\Sigma_t^{mi} + \Sigma_t^{li}\Sigma_t^{ms}) + O(\epsilon^2), \end{aligned} \tag{S24}$$

where δ_{ij} is the Kronecker delta function with value 1 when $i = j$ and 0 when $i \neq j$. Through different initial points, we can obtain all the components $(\boldsymbol{\mu}_{(k)}, \Sigma_{(k)})$ by finding the stable points

of the determined system

$$\begin{cases} \dfrac{\mathrm{d}\mu_t^i}{\mathrm{d}t} = L_1^i(\boldsymbol{\mu}_t, \Sigma_t, \epsilon) = b^i(\boldsymbol{\mu}_t) + \dfrac{\epsilon}{2}\nabla^2 b^i(\boldsymbol{\mu}_t) : \Sigma_t, \\ \dfrac{\mathrm{d}\Sigma_t^{ij}}{\mathrm{d}t} = L_2^{ij}(\boldsymbol{\mu}_t, \Sigma_t, \epsilon) \\ \qquad = \displaystyle\sum_k \partial_k b^i(\boldsymbol{\mu}_t)\Sigma_t^{kj} + \sum_k \partial_k b^j(\boldsymbol{\mu}_t)\Sigma_t^{ki} + 2\delta_{ij} \\ \qquad\quad + \dfrac{\epsilon}{6}\displaystyle\sum_{l,m,s} \partial^3_{lms} b^i(\boldsymbol{\mu}_t) \cdot (\Sigma_t^{lm}\Sigma_t^{sj} + \Sigma_t^{ls}\Sigma_t^{mj} + \Sigma_t^{lj}\Sigma_t^{ms}) \\ \qquad\quad + \dfrac{\epsilon}{6}\displaystyle\sum_{l,m,s} \partial^3_{lms} b^j(\boldsymbol{\mu}_t) \cdot (\Sigma_t^{lm}\Sigma_t^{si} + \Sigma_t^{ls}\Sigma_t^{mi} + \Sigma_t^{li}\Sigma_t^{ms}), \end{cases} \tag{S25}$$

where $L_1 = (L_1^i)_{n\times 1}$ is the evolving operator for $\boldsymbol{\mu}_t$, and $L_2 = (L_2^{ij})_{n\times n}$ is the evolving operator for Σ_t.

2. *Long time asymptotics*

To determine the mixture weights $\{\rho^{(k)}\}$, we need to consider the transitions between attractive basins in the long time scale $\log(t) \gtrsim O(1/\epsilon)$. In this regime, $\boldsymbol{X}_t$ is upscaled to a continuous-time Markov chain, which transits between different basins. Denote the Taylor expansion of $R(\boldsymbol{x})$ as

$$\begin{aligned} R(\boldsymbol{x}) =& R(\boldsymbol{\mu}_t) + \nabla R(\boldsymbol{\mu}_t)\cdot(\boldsymbol{x}-\boldsymbol{\mu}_t) + \frac{1}{2}\nabla^2 R(\boldsymbol{\mu}_t) : (\boldsymbol{x}-\boldsymbol{\mu}_t)^{(2)} \\ &+ \frac{1}{3!}\nabla^3 R(\boldsymbol{\mu}_t) : (\boldsymbol{x}-\boldsymbol{\mu}_t)^{(3)} + \frac{1}{4!}\nabla^4 R(\boldsymbol{\mu}_t) : (\boldsymbol{x}-\boldsymbol{\mu}_t)^{(4)} + o(||\boldsymbol{x}-\boldsymbol{\mu}_t||^4), \end{aligned} \tag{S26}$$

then the average BDR for state k is

$$R_k = \int R(x)\widehat{p}_{(k)}(\boldsymbol{x}, \boldsymbol{\mu}_{(k)}, \Sigma_{(k)})\,\mathrm{d}\boldsymbol{x} \approx R(\boldsymbol{\mu}_{(k)}) + \frac{\epsilon}{2}\nabla^2 R(\boldsymbol{\mu}_{(k)}) : \Sigma_{(k)}. \tag{S27}$$

Let $\boldsymbol{Q}$ be the upscaled generator matrix, in which Q_{ij} is the transition rate from state i to state j when $i \neq j$, and $Q_{ii} = -\sum_{j\neq i} Q_{ij}$. We can have the asymptotics for mixture weights as

$$\frac{\mathrm{d}\boldsymbol{\rho}_U}{\mathrm{d}t} = \boldsymbol{Q}^T\boldsymbol{\rho}_U + \boldsymbol{R}\boldsymbol{\rho}_U, \quad \frac{\mathrm{d}\boldsymbol{\rho}_0}{\mathrm{d}t} = \boldsymbol{Q}^T\boldsymbol{\rho}_0, \tag{S28}$$

where $\boldsymbol{R} = \mathrm{diag}(R_k)$, $\boldsymbol{\rho}_U = (\rho_U^{(k)})$ contains the mixture weights for $P_U(\boldsymbol{x})$, and $\boldsymbol{\rho}_0 = (\rho_0^{(k)})$ contains the mixture weights for $P_0(\boldsymbol{x})$.

The accurate $\boldsymbol{Q}$ is difficult to be obtained, so we use the approximation

$$\rho_0^{(k)} \approx \lim_{t\to\infty} \mathbb{E}\big[\delta(\boldsymbol{X}_t(\omega) \in \Omega_k)\big], \tag{S29}$$

where Ω_k denotes the k-th stable state, and $\boldsymbol{X}_0(\omega)$ is sampled under a uniform distribution on a finite domain. The approximation Eq. S29 can be taken as the percentage of trajectories falling into the stable state k (size of the basin).

To estimate the weight $\rho_U^{(k)}$, we denote $T_k^i \triangleq [a_i^{(k)}, b_i^{(k)}]$ as the time interval where $\boldsymbol{X}_t(\omega) \in \Omega_k$ when $t \in T_k^i, i \in \mathbb{N}_+$. The trajectory ω transits into Ω_k at $a_i^{(k)}$, exits Ω_k at $b_i^{(k)}$, and the holding

time is $h_i^{(k)} = b_i^{(k)} - a_i^{(k)}$. Set $q_k = -Q_{kk}$ as the exit rate of state k, then h_i obeys the exponential distribution with rate parameter q_k.

Then, by ergodic theorem we can obtain

$$\begin{aligned}
\rho_U^{(k)} &= \mathbb{E}\big[\rho_\infty(\omega)\delta(\boldsymbol{X}_\infty(\omega) \in \Omega_k)\big] = \lim_{T\to\infty} \frac{1}{T}\int_0^T \rho_t(\omega)\delta(\boldsymbol{X}_t(\omega) \in \Omega_k)\,\mathrm{d}t \\
&= \lim_{T\to\infty} \frac{M}{T}\cdot\frac{1}{M}\sum_{i=1}^{M}\int_{T_k^i} \rho_t(\omega)\,\mathrm{d}t = \lim_{T\to\infty} \frac{M}{T}\cdot\frac{1}{M}\sum_{i=1}^{M}\int_{T_k^i} \exp\Big(\int_0^t R(\boldsymbol{X}_s)\,\mathrm{d}s\Big)\,\mathrm{d}t \\
&= \lim_{T\to\infty} \frac{M}{T}\cdot\frac{1}{M}\sum_{i=1}^{M}\left\{\exp\Big(\int_0^{a_i^{(k)}} R(\boldsymbol{X}_s)\,\mathrm{d}s\Big)\cdot\int_{T_k^i}\exp\Big(\int_{a_i^{(k)}}^t R(\boldsymbol{X}_s)\,\mathrm{d}s\Big)\,\mathrm{d}t\right\}, \qquad \text{(S30)}
\end{aligned}$$

where M is the number of T_k^i in $[0,T]$. To ensure the non-explosion of the probability, we must assume that $\lim_{t\to\infty}\frac{1}{t}\int_0^t R(\boldsymbol{X}_s)\,\mathrm{d}s = \mathbb{E}R(\boldsymbol{X}_\infty) = 0$, and $\exp\Big(\int_0^{a_i^{(k)}} R(\boldsymbol{X}_s)\,\mathrm{d}s\Big) \approx 1$ when $a_i^{(k)}$ is sufficiently large. As M/T is the frequency of exiting Ω_k during $[0,T]$, we have $\lim_{T\to\infty} M/T = \rho_0^{(k)} q_k$. Thus, we have the estimation

$$\begin{aligned}
\rho_U^{(k)} &\approx \lim_{M\to\infty} \rho_0^{(k)} q_k \cdot \frac{1}{M}\sum_{i=1}^{M}\int_{T_k^i}\exp\Big(R_k(t-a_i^{(k)})\Big)\,\mathrm{d}t = \rho_0^{(k)} q_k\cdot\mathbb{E}_h\int_0^h \exp\Big(tR_k\Big)\,\mathrm{d}t \\
&= \rho_0^{(k)} q_k\int_0^\infty\left\{q_k\exp(-xq_k)\cdot\int_0^x\exp(tR_k)\,\mathrm{d}t\right\}\mathrm{d}x \\
&= \frac{\rho_0^{(k)} q_k}{q_k - R_k}, \qquad \text{(S31)}
\end{aligned}$$

where h is the random variable standing for the holding time, and $q_k \geqslant R_k$ to ensure the non-explosion of pdf.

Further, we assume the exit time τ_k for each component in $\widehat{F}(\boldsymbol{x})$ is in proportion to $\rho_0^{(k)}$. By $\tau_k = 1/q_k = C\rho_0^{(k)}$, where C is a constant, we can obtain

$$\rho_U^{(k)} \approx \frac{\rho_0^{(k)}}{1 - CR_k\rho_0^{(k)}}. \qquad \text{(S32)}$$

The constant C can be obtained by optimizing

$$\begin{aligned}
\min_{C\geqslant 0} \quad & \left\{\Big|\sum_{k=1}^{K}\frac{\rho_0^{(k)}R_k}{1 - CR_k\rho_0^{(k)}}\Big|^2\right\}, \\
\text{s.t.} \quad & CR_k\rho_0^{(k)} \leqslant 1, \quad k = 1,2,\ldots,K,
\end{aligned} \qquad \text{(S33)}$$

which is induced from the steady constraint $\int R(\boldsymbol{x})P_U(\boldsymbol{x})\,\mathrm{d}\boldsymbol{x} = 0$. $\rho_U^{(k)}$ is then normalized to ensure $\sum_{k=1}^K \rho_U^{(k)} = 1$.

After we have normalized $\rho_0^{(k)}$ and $\rho_U^{(k)}$, the steady pdfs $\widehat{P}_0(\boldsymbol{x})$ and $\widehat{P}_U(\boldsymbol{x})$ can be estimated by Eq. S19. Further, landscapes $\widehat{U}(\boldsymbol{x})$ and $\widehat{V}(\boldsymbol{x})$ are obtained by Eq. S3 and Eq. S4, respectively.

B. MFA for the population-level equation

There is MFA directly applied to the population-level equation Eq. S1, too. The short time asymptotics for the Gaussian components is

$$\begin{aligned}\frac{\mathrm{d}\mu_t^i}{\mathrm{d}t} &= \frac{\mathrm{d}}{\mathrm{d}t}\int x_i p(\boldsymbol{x},t)\,\mathrm{d}\boldsymbol{x} = \int x_i\cdot[-\nabla\cdot(\boldsymbol{b}(\boldsymbol{x})p(\boldsymbol{x},t))+\epsilon\Delta p(\boldsymbol{x},t)+R(\boldsymbol{x})p(\boldsymbol{x},t)]\,\mathrm{d}\boldsymbol{x}\\ &= b^i(\boldsymbol{\mu}_t)+\mu_t^i R(\boldsymbol{\mu}_t)+\frac{\epsilon}{2}\nabla^2 b^i(\boldsymbol{\mu}_t):\Sigma_t+\frac{\epsilon}{2}\mu_t^i\cdot(\nabla^2 R(\boldsymbol{\mu}_t):\Sigma_t)+\epsilon\cdot(\nabla R(\boldsymbol{\mu}_t)\cdot\Sigma_t^{\cdot i})\\ &\quad +O(\epsilon^2),\end{aligned} \tag{S34}$$

and

$$\begin{aligned}\frac{\mathrm{d}\Sigma_t^{ij}}{\mathrm{d}t} &= \frac{\mathrm{d}}{\mathrm{d}t}\int\frac{1}{\epsilon}(x_i-\mu_t^i)(x_j-\mu_t^j)p(\boldsymbol{x},t)\,\mathrm{d}\boldsymbol{x}\\ &= -\frac{1}{\epsilon}\int\frac{\mathrm{d}\mu_t^i}{\mathrm{d}t}\cdot(x_j-\mu_t^j)p(\boldsymbol{x},t)\,\mathrm{d}\boldsymbol{x}-\frac{1}{\epsilon}\int\frac{\mathrm{d}\mu_t^j}{\mathrm{d}t}\cdot(x_i-\mu_t^i)p(\boldsymbol{x},t)\,\mathrm{d}\boldsymbol{x}+\frac{1}{\epsilon}\int(x_i-\mu_t^i)(x_j-\mu_t^j)\frac{\partial p(\boldsymbol{x},t)}{\partial t}\,\mathrm{d}\boldsymbol{x}\\ &= 0+0+\frac{1}{\epsilon}\int(x_i-\mu_t^i)(x_j-\mu_t^j)[-\nabla\cdot(\boldsymbol{b}(\boldsymbol{x})p(\boldsymbol{x},t))+\epsilon\Delta p(\boldsymbol{x},t)+R(\boldsymbol{x})p(\boldsymbol{x},t)]\,\mathrm{d}\boldsymbol{x}\\ &= \sum_k\partial_k b^i(\boldsymbol{\mu}_t)\Sigma_t^{kj}+\sum_k\partial_k b^j(\boldsymbol{\mu}_t)\Sigma_t^{ki}+2\delta_{ij}+R(\boldsymbol{\mu}_t)\Sigma_t^{ij}\\ &\quad+\frac{\epsilon}{6}\sum_{l,m,s}\partial^3_{lms}b^i(\boldsymbol{\mu}_t)\cdot\int\frac{(x_l-\mu_t^l)(x_m-\mu_t^m)(x_s-\mu_t^s)(x_j-\mu_t^j)}{\epsilon^2}p(\boldsymbol{x},t)\,\mathrm{d}\boldsymbol{x}\\ &\quad+\frac{\epsilon}{6}\sum_{l,m,s}\partial^3_{lms}b^j(\boldsymbol{\mu}_t)\cdot\int\frac{(x_l-\mu_t^l)(x_m-\mu_t^m)(x_s-\mu_t^s)(x_i-\mu_t^i)}{\epsilon^2}p(\boldsymbol{x},t)\,\mathrm{d}\boldsymbol{x}\\ &\quad+\frac{\epsilon}{2}\sum_{l,m}\partial^2_{lm}R(\boldsymbol{\mu})\int\frac{(x_l-\mu_t^l)(x_m-\mu_t^m)(x_i-\mu_t^i)(x_j-\mu_t^j)}{\epsilon^2}p(\boldsymbol{x},t)\,\mathrm{d}\boldsymbol{x}+O(\epsilon^2).\end{aligned} \tag{S35}$$

For the Gaussian case, the forth order moments can be calculated by

$$\mathbb{E}_{p(\boldsymbol{x})}\left\{\frac{(x_l-\mu^l)(x_m-\mu^m)(x_s-\mu^s)(x_k-\mu^k)}{\epsilon^2}\right\}=\Sigma^{lm}\Sigma^{sk}+\Sigma^{ls}\Sigma^{mk}+\Sigma^{lk}\Sigma^{ms}. \tag{S36}$$

The estimations for weights are

$$\rho_0^{(k)}\approx\lim_{t\to\infty}\mathbb{E}\big[\delta(\boldsymbol{X}_t(\omega)\in\Omega_k^0)\big], \tag{S37}$$

and

$$\rho_U^{(k)}\approx\lim_{t\to\infty}\mathbb{E}\big[\rho_t(\omega)\delta(\boldsymbol{X}_t(\omega)\in\Omega_k^U)\big], \tag{S38}$$

where $\boldsymbol{X}_t(\omega)$ follows Eq. S10, Ω_k^0 is the kth state for $\widehat{P}_0$, and Ω_k^U is the kth state for $\widehat{P}_U$.

However, $\boldsymbol{\mu}_t$ and Σ_t depends on $R(\boldsymbol{x})$ now. When the system is with or without $R(\boldsymbol{x})$, the approximated global pdf Eq. S19 may have quite different number and position of the components (i.e. $\widehat{P}_0(\boldsymbol{x})$ and $\widehat{P}_U(\boldsymbol{x})$ can have disparate components). The severe consequence is that the energy landscape $V(\boldsymbol{x})$ could have a very bad performance to describe the differentiation after so many procedures with approximation. That's the reason why we propose MFA to Eq. S10 for multi-state systems in the applications.

But, for the system with only one state/component, Eq. S34 and Eq. S35 could differ $\widehat{P}_0$ and $\widehat{P}_U$, while the MFA to the weighted dynamics Eq. S25 fails.

C. Analytic solution of the mean-field approximation for two examples

In the MFA, we expand the dynamics $\boldsymbol{b}(\boldsymbol{x})$ and $R(\boldsymbol{x})$ to the order $O(\epsilon)$, instead of $O(1)$ which is used in reference [2]. We will use two analytic examples in this subsection to show its necessity, in order to get an accurate approximation to the landscape $V(\boldsymbol{x})$. An intuitive illustration in the gradient system is given by Eq. S8, which says

$$-\frac{1}{\epsilon}\nabla U(\boldsymbol{x})\cdot\nabla V(\boldsymbol{x})+\Delta V(\boldsymbol{x})=-R(\boldsymbol{x}). \tag{S39}$$

Taking $U(\boldsymbol{x})\sim O(1)$ $R(\boldsymbol{x})\sim O(1)$ for an example, we obtain $V(\boldsymbol{x})\sim O(\epsilon)$ and the approximation to the dynamics of P_0 and P_U needs to be at least $O(\epsilon)$.

1. 1-dim gradient system

In the first example, we use a one dimensional gradient system with true potentials $U(x)=\frac{1}{2}x^2$, $V(x)=\frac{\epsilon}{2}x^2$, and $F(x)=U(x)+V(x)=\frac{1+\epsilon}{2}x^2$. The task is to decompose the system with known $b(x)=-F'(x)=-(1+\epsilon)x$, and BDR $R(x)=\frac{1}{\epsilon}U'(x)V'(x)-V''(x)=x^2-\epsilon$.

The mean-field approximation to $\frac{\partial p(x,t)}{\partial t}=-\nabla\cdot(b(x)p(x,t))+\epsilon\Delta p(x,t)+R(x)p(x,t)$ by Eq. S34 and Eq. S35 is

$$\begin{cases}\frac{\mathrm{d}\mu_t}{\mathrm{d}t}=b(\mu_t)+\mu_tR(\mu_t)+\epsilon\Sigma_tR'(\mu_t)+\frac{\epsilon}{2}\mu_tR''(\mu_t)\Sigma_t,\\ \frac{\mathrm{d}\Sigma_t}{\mathrm{d}t}=2b'(\mu_t)\Sigma_t+2+R(\mu_t)\Sigma_t+\frac{3\epsilon}{2}R''(\mu_t)\Sigma_t^2,\end{cases}\xrightarrow{t\to+\infty}\begin{cases}\mu_\infty(\mu_\infty^2-2\epsilon-1+3\epsilon\Sigma_\infty)=0,\\ 3\epsilon\Sigma_\infty^2+(\mu_\infty^2-3\epsilon-2)\Sigma_\infty+2=0.\end{cases} \tag{S40}$$

We can find that only $\mu_\infty=0,\Sigma_\infty=1$ is the stable solution, and the approximated steady pdf is $\widehat{P}_U(x)=\frac{1}{Z_U}\exp\left(-\frac{1}{2\epsilon}x^2\right)$, where Z_U is a normalization constant. The estimated energy landscape U by Eq. S3 is $\widehat{U}(x)=\frac{1}{2}x^2+\epsilon\log(Z_U)$, which equals to $U(x)$ in the sense of ignoring a constant.

The mean-field approximation to $\frac{\partial p(x,t)}{\partial t}=-\nabla\cdot(b(x)p(x,t))+\epsilon\Delta p(x,t)$ is

$$\begin{cases}\frac{\mathrm{d}\mu_t}{\mathrm{d}t}=b(\mu_t),\\ \frac{\mathrm{d}\Sigma_t}{\mathrm{d}t}=2b'(\mu_t)\Sigma_t+2,\end{cases}\xrightarrow{t\to+\infty}\begin{cases}-(1+\epsilon)\mu_\infty=0,\\ -2(1+\epsilon)\Sigma_\infty+2=0,\end{cases}\Rightarrow\begin{cases}\mu_\infty=0,\\ \Sigma_\infty=\frac{1}{1+\epsilon}.\end{cases} \tag{S41}$$

We can get the approximated steady pdf $\widehat{P}_0(x)=\frac{1}{Z_0}\exp\left(-\frac{1+\epsilon}{2\epsilon}x^2\right)$, where Z_0 is a normalization constant. Further, the estimated energy landscape $V(x)$ by Eq. S4 is $\widehat{V}(x)=\frac{\epsilon}{2}x^2-\epsilon\log(Z_U/Z_0)$, which equals to $V(x)$ in the sense of ignoring a constant.

Thus, in this example, we show that the mean-field approximation can get the correct decomposition of the system for a one-dimensional Ornstein-Uhlenback process with source and sink/birth and death. As a remark, if the approximations in Eq. S34 and Eq. S35 are not in the order of $O(\epsilon)$ but only $O(1)$, the estimation of P_U in Eq. S40 will be incorrect and we may obtain spurious $\widehat{U}(x),\widehat{V}(x)$.

2. 2-dim non-gradient system

In the second example, we use a two dimensional non-gradient system with true terms $U(\boldsymbol{x})=\frac{1}{2}(x_1^2+x_2^2)$, $V(\boldsymbol{x})=\frac{\epsilon}{2}(x_1^2+x_2^2)$, $\boldsymbol{f}(\boldsymbol{x})=Z_0\exp\left(\frac{1+\epsilon}{2\epsilon}(x_1^2+x_2^2)\right)\cdot[-x_2,x_1]^T$, where $Z_0=\frac{2\pi\epsilon}{1+\epsilon}$. The

task is to decompose the system with known

$$\boldsymbol{b}(\boldsymbol{x}) = -(1+\epsilon)\begin{bmatrix} x_1 \\ x_2 \end{bmatrix} + Z_0 \mathrm{e}^{\frac{1+\epsilon}{2\epsilon}(x_1^2+x_2^2)} \begin{bmatrix} -x_2 \\ x_1 \end{bmatrix}, \quad \text{and} \quad R(\boldsymbol{x}) = (x_1^2+x_2^2) - 2\epsilon. \tag{S42}$$

By solving Eq. S34 and Eq. S35 at $t = +\infty$, we can obtain a stable point $\boldsymbol{\mu}_\infty = [0,0]^T$ and $\Sigma_\infty = I$ when the non-zero $R(\boldsymbol{x})$ exists, where I is the 2-dim unit matrix. Thus, the mean-field approximation of the steady pdf is $\widehat{P}_U(\boldsymbol{x}) = \frac{1}{2\pi\epsilon}\exp\left(-\frac{1}{2\epsilon}(x_1^2+x_2^2)\right)$. The energy landscape U is estimated by Eq. S3 as $\widehat{U}(\boldsymbol{x}) = \frac{1}{2}(x_1^2+x_2^2)+\epsilon\log(2\pi\epsilon)$, which equals to $U(\boldsymbol{x})$ in the sense of ignoring a constant.

When setting $R(\boldsymbol{x}) \equiv 0$, by solving Eq. S34 and Eq. S35 at $t = +\infty$, we can obtain a stable point $\boldsymbol{\mu}_\infty = [0,0]^T$ and $\Sigma_\infty = \frac{1}{1+\epsilon}I$. The mean-field approximation of the steady pdf is $\widehat{P}_0(\boldsymbol{x}) = \frac{1+\epsilon}{2\pi\epsilon}\exp\left(-\frac{1+\epsilon}{2\epsilon}(x_1^2+x_2^2)\right)$. The estimation of energy landscape $V(\boldsymbol{x})$ by Eq. S4 is $\widehat{V}(\boldsymbol{x}) = \frac{1}{2}(x_1^2+x_2^2) - \epsilon\log(1+\epsilon)$, which equals to $V(\boldsymbol{x})$ in the sense of ignoring a constant. Further, the curl flux $\boldsymbol{f}(\boldsymbol{x})$ is estimated by Eq. S5 as $\widehat{\boldsymbol{f}}(\boldsymbol{x}) = Z_0\exp\left(\frac{1+\epsilon}{2\epsilon}(x_1^2+x_2^2)\right)\cdot[-x_2, x_1]^T$, which is exactly the true $\boldsymbol{f}(\boldsymbol{x})$.

In this example, we show that the mean-field approximation can get the correct decomposition of the system for a two-dimensional non-gradient process with source and sink/birth and death. It can be also found that the expansions to the order $O(\epsilon)$ in Eq. S34 and Eq. S35 are necessary for the correct estimation of U, V and $\boldsymbol{f}$.

IV. DETAILS OF THE NUMERICAL EXAMPLES

A. Drift-diffusion process

The dynamics of the 2-dim drift-diffusion process is

$$\begin{cases} \dfrac{\mathrm{d}x_1}{\mathrm{d}t} = -(x_1^3 + x_1 x_2), \\ \dfrac{\mathrm{d}x_2}{\mathrm{d}t} = -(\dfrac{x_1^2}{2} + 2x_2^3 - \dfrac{3}{2}x_2), \end{cases} \tag{S43}$$

which is a gradient system with potential $F(\boldsymbol{x}) = \left(\frac{x_1^2}{2} + \frac{x_2}{2}\right)^2 + \frac{(x_2^2-1)^2}{2}$, and white noise with amplitude $\sqrt{2\epsilon}$ can be added to the system. $\epsilon = 0.01$. BDR is set as $R(\boldsymbol{x}) = 0.3[(x_1^2-1)^2 + (x_2+1)^2 - 1]$.

B. Two-gene fate decision

The dynamics for the two-gene fate decision system is

$$\begin{cases} \dfrac{\mathrm{d}x_1}{\mathrm{d}t} = \dfrac{\alpha_1 x_1^n}{S^n + x_1^n} + \dfrac{\beta_1 S^n}{S^n + x_2^n} - k_1 x_1 \\ \dfrac{\mathrm{d}x_2}{\mathrm{d}t} = \dfrac{\alpha_2 x_2^n}{S^n + x_2^n} + \dfrac{\beta_2 S^n}{S^n + x_1^n} - k_2 x_2, \end{cases} \tag{S44}$$

where the two genes x_1 and x_2 have self-activation and inter-inhibition, white noise with amplitude $\sqrt{2\epsilon}$ can be added to the system, and parameters are set as $\alpha_1 = \alpha_2 = 0.3$, $\beta_1 = \beta_2 = 0.5$, $n = 4$,

$k_1 = k_2 = 1$, and $S = 0.5$. $\epsilon = 0.01$. BDR is set as $R(\boldsymbol{x}) = -r[(x_1-1)^2 + (x_2-1)^2 - 0.5]$, where the rate amplitude r can vary from 0 to 30.

C. T-cell differentiation process

The dynamics for the T-cell differentiation [6] is modeled as

$$\begin{cases} \dfrac{\mathrm{d}x_1}{\mathrm{d}t} = k_{0T} + K_T \cdot \dfrac{x_1^{n_{TT}}}{x_1^{n_{TT}} + K_{TT}^{n_{TT}}} \cdot \dfrac{x_3^{n_{TG}}}{x_3^{n_{TG}} + K_{TG}^{n_{TG}}} \cdot \dfrac{K_{TP}^{n_{TP}}}{x_2^{n_{TP}} + K_{TP}^{n_{TP}}} + k_{TN} \cdot \alpha_N - \gamma_T \cdot x_1 \\ \dfrac{\mathrm{d}x_2}{\mathrm{d}t} = k_{0P} + K_P \cdot \dfrac{x_2^{n_{PP}}}{x_2^{n_{PP}} + K_{PP}^{n_{PP}}} \cdot \dfrac{K_{PG}^{n_{PG}}}{x_3^{n_{PG}} + K_{PG}^{n_{PG}}} \cdot \dfrac{K_{PB}^{n_{PB}}}{x_4^{n_{PB}} + K_{PB}^{n_{PB}}} \cdot \dfrac{K_{PT}^{n_{PT}}}{x_1^{n_{PT}} + K_{PT}^{n_{PT}}} - \gamma_P \cdot x_2 \\ \dfrac{\mathrm{d}x_3}{\mathrm{d}t} = k_{0G} + K_G \cdot \dfrac{x_1^{n_{GT}}}{x_1^{n_{GT}} + K_{GT}^{n_{GT}}} \cdot \dfrac{K_{GP}^{n_{GP}}}{x_2^{n_{GP}} + K_{GP}^{n_{GP}}} + k_{GN} \cdot \alpha_N - \gamma_G \cdot x_3 \\ \dfrac{\mathrm{d}x_4}{\mathrm{d}t} = k_{0B} + K_B \cdot \dfrac{x_1^{n_{BT}}}{x_1^{n_{BT}} + K_{BT}^{n_{BT}}} \cdot \dfrac{x_3^{n_{BG}}}{x_3^{n_{BG}} + K_{BG}^{n_{BG}}} + k_{BN} \cdot \alpha_N - \gamma_B \cdot x_4, \end{cases} \tag{S45}$$

where variables are x_1 : TCF-1, x_2 : PU.1, x_3 : GATA3, x_4 : BCL11B, white noise with amplitude $\sqrt{2\epsilon}$ can be added to the system, and parameters are

$$\begin{aligned} & k_{0T} = 0.48, K_T = 1.12, n_{TT} = 4, K_{TT} = 0.5, n_{TG} = 6, K_{TG} = 0.4, n_{TP} = 6, K_{TP} = 3.4, k_{TN} = 1, \gamma_T = 1, \\ & k_{0P} = 0.32, K_P = 2.88, n_{PP} = 6, K_{PP} = 0.78, n_{PG} = 4, K_{PG} = 2.5, n_{PB} = 3, K_{PB} = 2.6, n_{PT} = 2, K_{PT} = 2.5, \gamma_P = 1, \\ & k_{0G} = 0, K_G = 2,2, n_{GT} = 2, K_{GT} = 0.2, n_{GP} = 6, K_{GP} = 2,2, k_{GN} = 0.02, \gamma_G = 1, \\ & k_{0B} = 0, K_B = 5.5, n_{BT} = 2, K_{BT} = 1.9, n_{BG} = 6, K_{BG} = 1.5, k_{BN} = 0.06, \gamma_B = 1, \\ & \alpha_N = 0. \end{aligned}$$

The Hill function $\frac{x^n}{x^n+K^n}$ represents for activation and $\frac{K^n}{x^n+K^n}$ stands for inhibition. Notch signal α_N, which regulates x_1, x_3 and x_4, is fixed as zero in our simulation. $\epsilon = 0.001$. BDR is set as $R(\boldsymbol{x}) = 30[4.2 - x_1^2 - (x_2-4)^2]$.

We use the mean-field approximation Eq. S25 to calculate the ELD. In order to simplify the computing complexity, we only consider diagonal elements of Σ_t, and Eq. S25 can be reduced to

$$\begin{cases} \dfrac{\mathrm{d}\mu_t^i}{\mathrm{d}t} = b^i(\boldsymbol{\mu}_t) + \dfrac{\epsilon}{2} \displaystyle\sum_{j=1}^{4} \partial_{jj}^2 b^i(\boldsymbol{\mu}_t) \cdot \Sigma_t^{jj}, \\ \dfrac{\mathrm{d}\Sigma_t^{ii}}{\mathrm{d}t} = 2\partial_i b^i(\boldsymbol{\mu}_t) \cdot \Sigma_t^{ii} + 2 + \epsilon \displaystyle\sum_{j=1}^{4} \partial_{jji} b^i(\boldsymbol{\mu}_t) \cdot \Sigma_t^{ii} \cdot \Sigma_t^{jj}, \\ \mathrm{d}\rho_t(\omega) = R(\boldsymbol{X}_t(\omega))\rho_t(\omega)\,\mathrm{d}t, \\ R_k = R(\boldsymbol{\mu}_{(k)}) + \dfrac{\epsilon}{2} \displaystyle\sum_{j=1}^{4} \partial_{jj} R(\boldsymbol{\mu}_{(k)}) \cdot \Sigma_{(k)}^{jj}, \quad \text{when } \boldsymbol{X}_t(\omega) \text{ in state } k. \end{cases} \tag{S46}$$

D. 10-dimensional system with the birth and death term

We use 10-dim dynamics to show that the MFA is applicable to the ELD in high-dimensional space with

$$\mathrm{d}\boldsymbol{X}(t) = -\nabla F(\boldsymbol{X}(t))\,\mathrm{d}t + \sqrt{2\epsilon}\,\mathrm{d}\boldsymbol{W}(t), \tag{S47}$$

where $\boldsymbol{X}(t) \in \mathbb{R}^{10}$, the potential function is

$$F(\boldsymbol{X}) = 10^{-4} \cdot \left[\sum_{i=1}^{10}(X_i - 1)^2\right] \cdot \left[\sum_{i=1}^{10} X_i^2\right] \cdot \left[\sum_{i=1}^{10}(X_i + 1)^2\right], \tag{S48}$$

noise amplitude is $\epsilon = 10^{-4}$, and $\boldsymbol{W}(t)$ is the standard Brownian motion.

There are three metastable states around $(1, 1, \dots, 1)^T$, $(-1, -1, \dots, -1)^T$, and $(0, 0, \dots, 0)^T$, respectively. We use a BDR term

$$R(\boldsymbol{X}) = \sum_{i=1}^{10} X_i^3. \tag{S49}$$

When we use the MFA Eq. S25 to calculate the ELD, the results of landscapes U and V are shown in Fig. S2. U can indicate the metastable states, while V can imply the pluripotency or differentiation direction successfully.

---

[1] J. Wang, C. Li, and E. Wang, Proc Natl Acad Sci USA **107**, 8195 (2010).
[2] J. Wang, K. Zhang, L. Xu, and E. Wang, Proc Natl Acad Sci USA **108**, 8257 (2011).
[3] C. Weinreb, S. Wolock, B. K. Tusi, M. Socolovsky, and A. M. Klein, Proc Natl Acad Sci USA **115**, E2467 (2018).
[4] J. Shi, T. Li, L. Chen, and K. Aihara, PLoS Comput Biol **15**, e1007488 (2019).
[5] C. Johnson, *Numerical solution of partial differential equations by the finite element method* (Courier Corporation, 2012).
[6] Y. Ye, X. Kang, J. Bailey, C. Li, and T. Hong, PLoS Comput Biol **15**, e1006855 (2019).

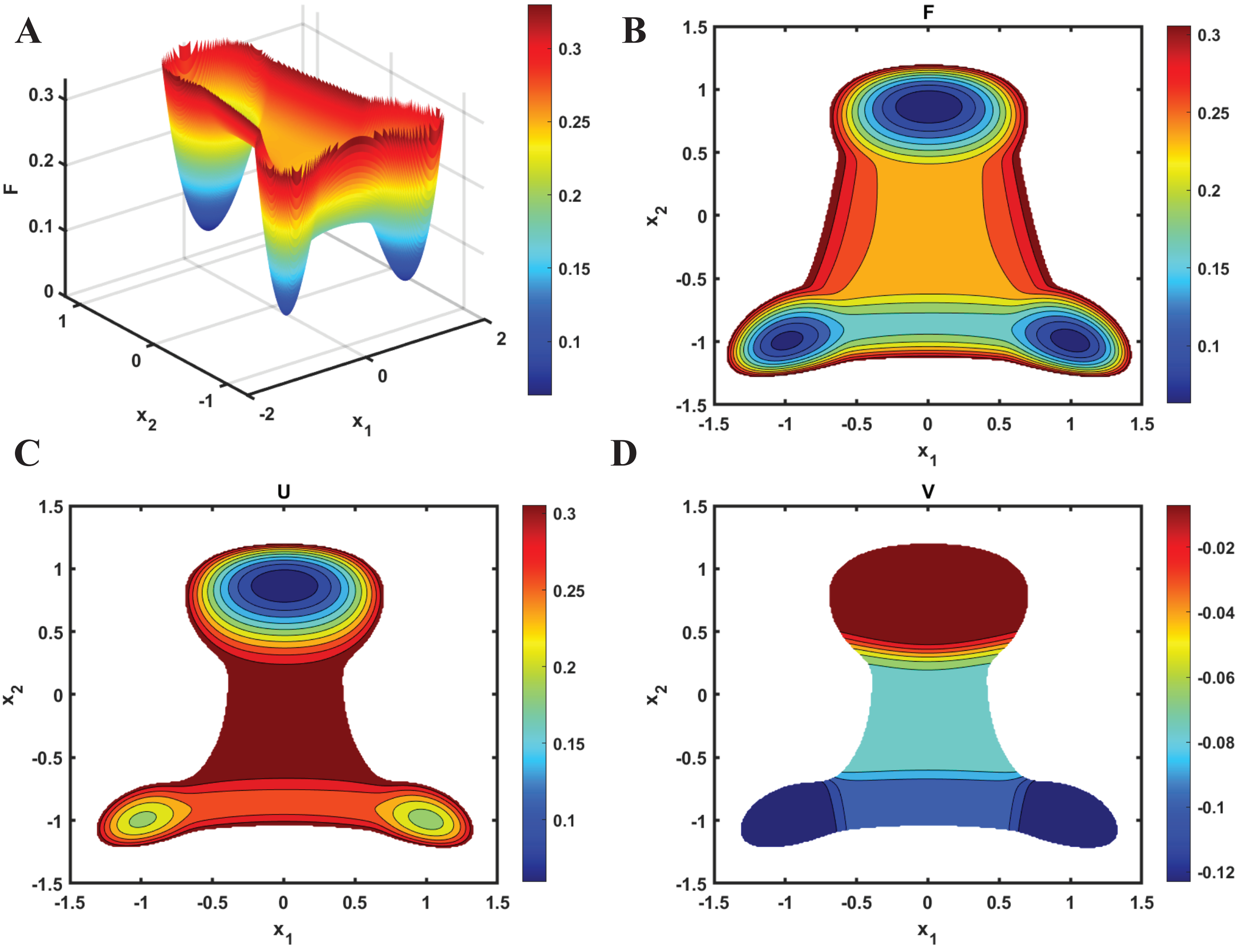

FIG. S1: Supplementary figures of the landscapes of the 2-dim drift-diffusion process. (A) shows the potential $F(\boldsymbol{x})$ in 3-dimensional space, and (B) is its projection in 2-dimensional space. (C) and (D) are the projections of landscapes U and V in the 2-dimensional space, respectively.

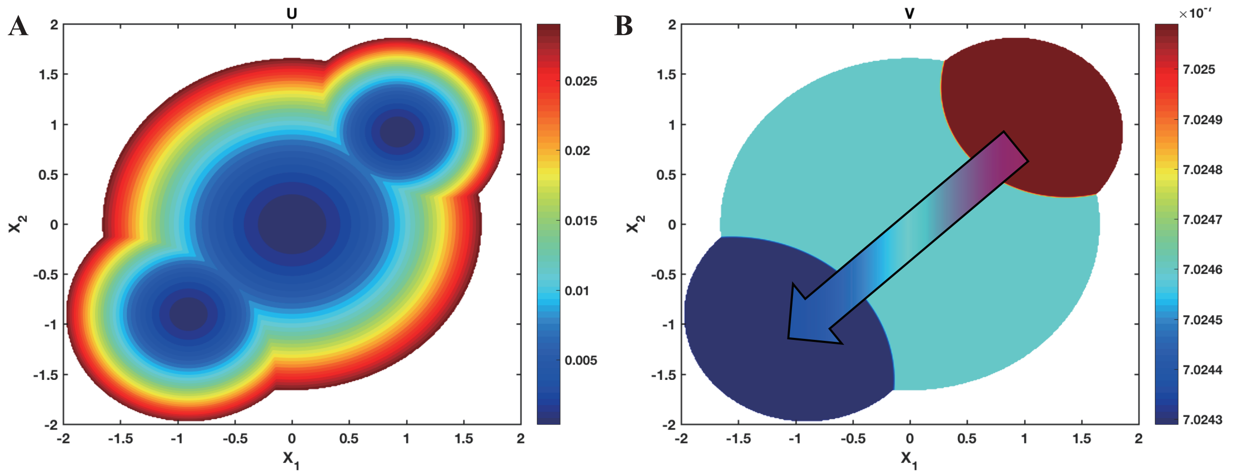

FIG. S2: Energy landscape decomposition of the 10-dimensional dynamical system. The landscapes U and V are approximated by the MFA and reduced to the first two variables' space. (A) shows the metastable landscape U, where there are three metastable states. (B) shows the pluripotency landscape V, which can indicate the differentiation direction from a high value to a low one. The differentiation direction is indicated by the illustrative arrow shown in (B).
